# New Dimeric and *seco*-Abietane Diterpenoids from *Salvia wardii*

**DOI:** 10.1007/s13659-015-0054-6

**Published:** 2015-04-08

**Authors:** Qiu-Li Xiao, Fan Xia, Xing-Wei Yang, Yang Liao, Li-Xin Yang, Yu-Kun Wei, Xian Li, Gang Xu

**Affiliations:** 1 School of Pharmaceutical Science and Yunnan Key Laboratory of Pharmacology of Natural Products, Kunming Medical University, Kunming, 650500 Yunnan People’s Republic of China; 2 State Key Laboratory of Phytochemistry and Plant Resources in West China, Kunming Institute of Botany, Chinese Academy of Sciences, Kunming, 650201 People’s Republic of China; 3 Shanghai Chenshan Plant Science Research Center, Chinese Academy of Sciences, Beijing, People’s Republic of China

**Keywords:** *Salvia wardii*, Dimeric abietane diterpenoids, *seco*-abietane

## Abstract

Two dimeric abietane diterpenoids, salviwardins A and B (**1** and **2**), and a *seco*-abietane diterpenoid salviwardin C (**3**), along with five known analogues (**4**–**8**), were isolated from the roots of *Salvia wardii.* The structures of these isolates were elucidated by extensive spectroscopic methods. The inhibitory activities of these isolates against five human cancer cell lines in vitro were also tested.

**Electronic supplementary material:**

The online version of this article (doi:10.1007/s13659-015-0054-6) contains supplementary material, which is available to authorized users.

## Introduction

The genus *Salvia* is a rich source of diterpenoids with structural diversity [1–4]. Hundreds of diterpenoids with interesting bioactivities, such as tanshinone IIA (treat cardiovascular diseases), salvicine (a significant antitumor agent), neotanshinlactone (inhibition of breast cancer), and salvinorin A (the first non-nitrogenated opium receptor agonist), have been characterized from the plants within this genus [5–8]. Many species of this genus, such as *S. miltiorrhiza*, *S. yunnanensis*, and *S. przewalskii*, are used to treat cardiovascular diseases [9–11], and *S. prionitis *is used in Chinese folk medicine for the treatment of tonsillitis, pharyngitis, and bacillary dysentery [12].

*Salvia wardii*, a herb with violet flowers distributed in east of Tibet, has not been chemically studied before [13]. Aiming at searching for structurally interesting and bioactive diterpenoids from the *Salvia* plants, we chemically investigated *S. wardii* and isolated three new abietane diterpenoids, salviwardins A**–**C (**1–3**), and five known analogues (**4–8**). The inhibitory activities of these isolates against five human cancer cell lines in vitro were also tested.

## Results and Discussion

The acetone extract of the air-dried and powdered the roots of *S. wardii* (33 kg) was subjected to a silica gel column to afford fractions A**–**G. Fraction B was subjected to a series of chromatographic methods, and led to the isolation of three new abietane derivatives, salviwardins A**–**C (**1–3**), together with five knows analogues, including prionitin (**4**) [14], sahandol (**5**) [15], salvilenone (**6**) [16], microstegiol (**7**) [17], and ferruginol (**8**) [18].

Salviwardin A (**1**) was obtained as orange powder. Its molecular formula C_40_H_54_O_4_ was established by its ^13^C NMR and HREIMS (*m/z* 598.4029, [M]^+^) data, indicating 14 degrees of unsaturation. The IR absorption at 3440 and 1624 cm^−1^ implied the existence of hydroxyl and carbonyl groups. The ^13^C and DEPT NMR (Table 1) spectroscopic data of **1** revealed 40 carbon signals, comprising fifteen quaternary carbons (one carbonyl, nine olefinic, and one oxygenated group), seven methines (one oxygenated and three olefinic ones), eight methylenes and ten methyls. The ^1^H NMR (Table 2) spectrum of **1** showed the presence of two isopropyl groups and six singlet methyls. The ^13^C and DEPT NMR spectroscopic data showed four noticeable quaternary signals for abietane diterpenoid at *δ*_C_ 40.1 (s, C-4), *δ*_C_ 46.7 (s, C-10), *δ*_C_ 33.8 (s, C-4′), *δ*_C_ 39.3 (s, C-10′) [19, 20]. These evidences indicated that compound **1** should be a dimer of two abietane diterpenoids units.

**Table 1:** ^13^C NMR data for **1** and **2** in CDCl_3_ (100 MHz, *δ* in ppm, *J* in Hz)

Position	**1**	**2**
1	31.7, CH_2_	31.7, CH_2_
2	18.5, CH_2_	18.5, CH_2_
3	38.9, CH_2_	38.8, CH_2_
4	40.1, C	40.1, C
5	80.2, C	82.7, C
6	70.0, CH	69.8, CH
7	141.2, CH	140.9, CH
8	140.4, C	133.9, C
9	124.7, C	124.5, C
10	46.7, C	46.7, C
11	144.1, C	144.2, C
12	181.8, C	181.8, C
13	141.3, C	141.5, C
14	135.0, CH	134.8, CH
15	26.9, CH	26.9, CH
16	21.5, CH_3_	21.7, CH_3_
17	21.0, CH_3_	21.5, CH_3_
18	24.3, CH_3_	24.2, CH_3_
19	30.8, CH_3_	30.9, CH_3_
20	24.2, CH_3_	24.3, CH_3_
1′	36.9, CH_2_	36.8, CH_2_
2′	19.4, CH_2_	19.3, CH_2_
3′	41.5, CH_2_	40.8, CH_2_
4′	33.8, C	33.3, C
5′	53.0, CH	51.6, CH
6′	19.3, CH_2_	126.9, CH
7′	32.4, CH_2_	128.0, CH
8′	127.2, C	125.8, C
9′	135.0, C	132.7, C
10′	39.3, C	41.3, C
11′	141.6, C	143.1, C
12′	133.8, C	139.5, C
13′	132.8, C	132.9, C
14′	119.5, CH	118.0, CH
15′	27.6, CH	27.3, CH
16′	22.3, CH_3_	22.5, CH_3_
17′	21.5, CH_3_	22.2, CH_3_
18′	33.9, CH_3_	33.0, CH_3_
19′	22.2, CH_3_	20.9, CH_3_
20′	20.4, CH_3_	18.3, CH_3_

**Table 2:** ^1^H NMR data for compounds **1** and **2** in CDCl_3_ (400 MHz, *δ* in ppm, *J* in Hz)

No.	**1**	**2**
1	2.42, td (5.1, 16.1)	2.40, td (5.5, 16.9,)
	2.79, m	2.77, br.d (16.9)
2	1.72, m	1.72, m
3	1.86, m	1.87, td (4.9, 15.9)
	1.29, overlap	1.29, m
6	5.42, d (3.4)	5.37, d (3.5)
7	6.07, d (3.4)	5.98, d (3.5)
14	6.55, s	6.51, s
15	2.95, sept (8.7)	2.95, sept (8.3)
16	1.03, d (8.7)	1.06, d (8.3)
17	1.07, d (8.7)	1.08, d (8.3)
18	1.32, s	1.32, s
19	1.02, s	1.05, s
20	1.40, s	1.39, s
1′	1.20, m	1.72, m
	3.11, br.d (16.3)	3.03, br.d (16.4)
2′	1.54, m	1.77, m
	1.79, m	1.61, m
3′	1.20, m	1.25, m
	1.45, m	1.47, br.d (17.3)
5′	1.26, m	2.14, t (3.7)
6′	1.79, m	5.82, dd (3.6, 11.9)
	1.54, m	
7′	2.75, m	6.37, dd (3.8, 11.9)
14′	6.36, s	6.41, s
15′	2.95, sept (8.5)	2.95, sept (8.6)
16′	0.84, d (8.5)	0.86, d (8.6)
17′	1.02, d (8.5)	1.04, d (8.6)
18′	0.95, s	1.07, s
19′	0.95, s	0.96, s
20′	1.43, s	1.18, s

**Fig. 1:**
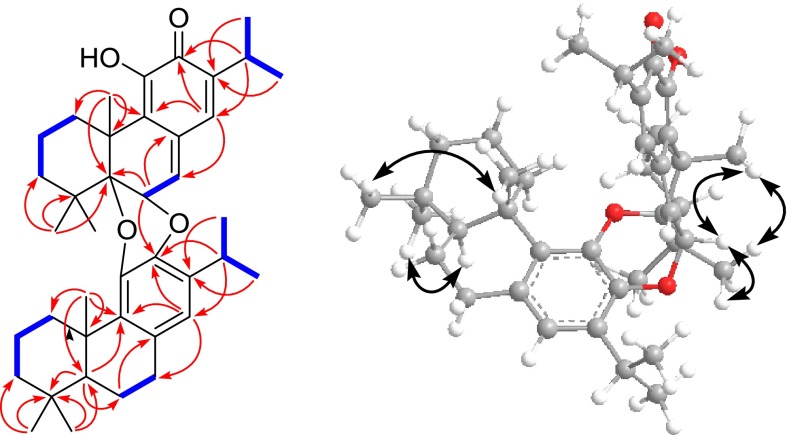
Key HMBC (), ^1^H-^1^H COSY (), and ROESY () correlations of **1**

Analysis of the 1D and 2D NMR spectra distinguished two sets of diterpenoid signals, C-1–C-20 and C-1′–C-20′, respectively. According to the characteristic signals for normal abietane diterpenoids at *δ*_C_ 40.1 (s, C-4), *δ*_C_ 46.7 (s, C-10), *δ*_C_ 24.3 (q, C-18), *δ*_C_ 30.8 (q, C-19), *δ*_C_ 24.2 (q, C-20), and an isopropyl group at *δ*_C_ 26.9 (d, C-15), *δ*_C_ 21.5 (q, Me-16), and *δ*_C_ 21.0 (q, Me-17), the structure of unit 1 can be ascribed to be an abietane diterpenoid [21–23]. The HMBC correlations (Fig. 1) from Me-20 (*δ*_H_ 1.40) to C-1 (*δ*_C_ 31.7), C-5 (*δ*_C_ 80.2), C-9 (*δ*_C_ 124.7), and C-10; from Me-18 (*δ*_H_ 1.32) and Me-19 (*δ*_H_ 1.02) to C-3 (*δ*_C_ 38.9), C-4, and C-5; from H-15 (*δ*_H_ 26.9) to C-12 (*δ*_C_ 181.8), C-13 (*δ*_C_ 141.3), and C-14 (*δ*_C_ 135.0); from H-14 (*δ*_H_ 6.55) to C-7 (*δ*_C_ 141.2), C-9, and C-12; and from H-6 (*δ*_H_ 5.42) to C-8 (*δ*_C_ 140.8), together with proton spin systems H-1/H-2/H-3 and H-6/H-7 obtained from the ^1^H–^1^H COSY spectrum (Fig. 1), established the structure of the unit 1.

The other unit was also deduced to be an abietane diterpenoid based on the characteristic quaternary signals at *δ*_C_ 33.8 (s, C-4′) and *δ*_C_ 39.3 (s, C-10′), the typical methyls at *δ*_C_ 33.9, *δ*_C_ 22.2, *δ*_C_ 20.4 for Me-18′, Me-19′, and Me-20′, respectively, and the isopropyal group at *δ*_C_ 27.6 (C-15′), *δ*_C_ 22.3 (Me-16′), and *δ*_C_ 21.5 (Me-17′). The planar structure of this unit was elucidated to be almost identical with that of the known analogue, dethdroabietane-11,12-diol [24], based on the comparative analysis of their NMR spectral data and the HMBC correlations from Me-20′ (*δ*_H_ 1.43) to C-1′ (*δ*_C_ 36.9), C-5′ (*δ*_C_ 53.0), C-9′ (*δ*_C_ 135.0), and C-10′; from Me-18′ (*δ*_H_ 0.95) and Me-19′ (*δ*_H_ 0.95) to C-3′, C-4′, and C-5′; from H-15′ (*δ*_H_ 27.6) to C-12′ (*δ*_C_ 133.8), C-13′ (*δ*_C_ 132.8), and C-14′ (*δ*_C_ 119.5); from H-14′ (*δ*_H_ 6.36) to C-7′ (*δ*_C_ 32.4), C-9′, and C-12′; and from H-5′ (*δ*_H_ 1.26) to C-4′ and C-6′ (*δ*_C_ 19.3), together with two proton spin systems, H-1′/H-2′/H-3′ and H-6′/H-7′, observed from the ^1^H–^1^H COSY spectrum (Fig. 1).

The two units account for 13 degrees of unsaturation. Since the totally degrees of unsaturation were 14, the remained one degree of unsaturation should be ascribed to the linkage between the two units through C-11/C-5/C-6 to C-11′/C-12′ to create an additional ring. The HMBC of H-6/C-12′ confirmed the linkage of C-6/C-12′ through an oxygen atom. In the ^1^H NMR spectrum of **1**, an obvious OH signal can be found at *δ*_H_ 7.50, and this OH group can be deduced to be attached at C-11 as evidenced by its HMBC correlations with C-9 (*δ*_C_ 124.7), C-11 (*δ*_C_ 144.1), and C-12 (*δ*_C_ 181.8). Then, the remained oxygenated quaternary at (C-5) and the oxygenated aromatic quaternary carbon (C-11′) were deduced to linked through ether bridge.

In the NOESY spectrum (Fig. 1), diagnostic cross-peaks of H-6/Me-20, Me-19/H-6, and Me-19/Me-20 indicated the *β*-orientation of H-6, Me-19, and Me-20. The NOE correlations of Me-20′/Me-19′ and Me-18′/H-5′ indicated that H-5′ was *α*-oriented. In addition, the *α*-substitution of O-5 was suggested by analysis of the molecular model of **1**, otherwise the NOE correlation of H-6/Me-19 should be unobservable.. Thus, the structure of **1** was elucidated and named salviwardin A.

**Fig. 2:**
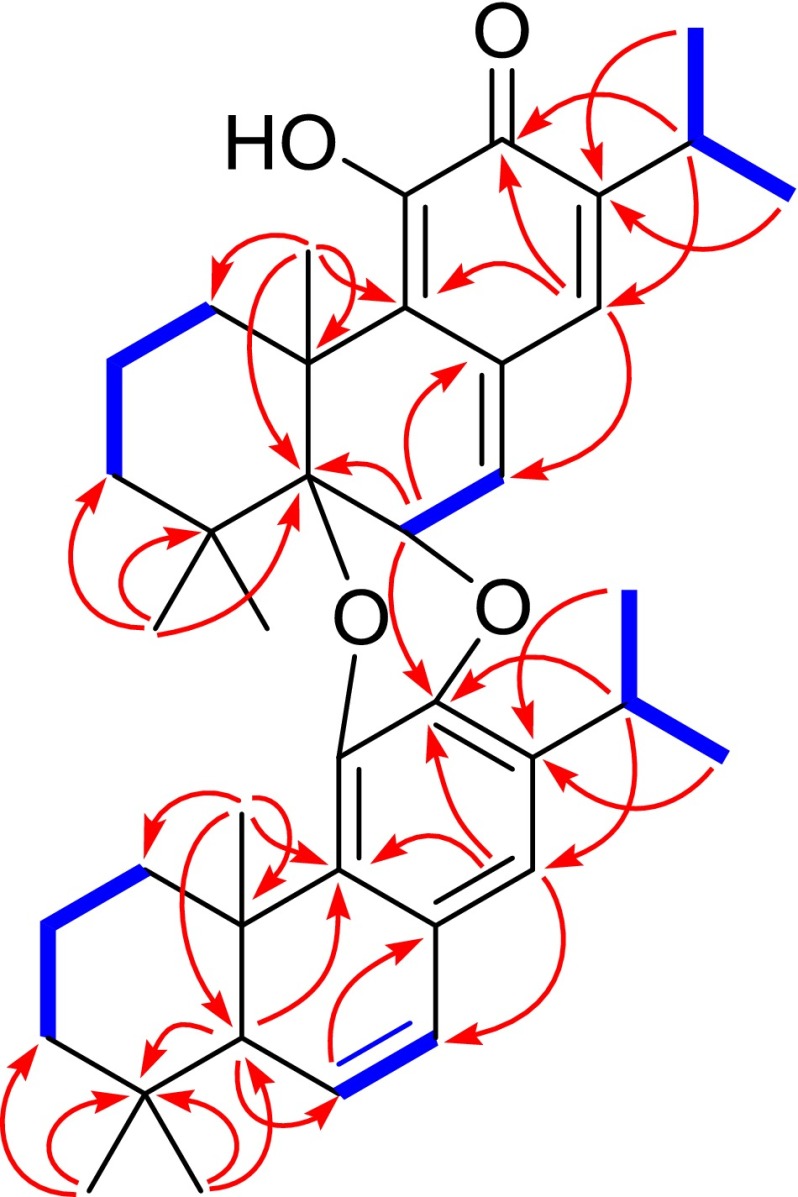
Key HMBC () and ^1^H-^1^H COSY () correlations of **2**

The molecular formula of salviwardin B (**2**) was determined to be C_40_H_52_O_4_ from its ^13^C NMR and HRESIMS spectral data, indicating one more unsaturation than **1**. Comparison of their 1D and 2D NMR data indicated that the structures of **1** and **2** were similar to each other (Tables 1, 2). The difference lied in that the two methylenes (C-6′ *δ*_C_ 19.3 and C-7′ *δ*_C_ 32.4) in **1** were replaced by a double bond (C-6′ *δ*_C_ 126.9 and C-7′ *δ*_C_ 128.0) in **2**, which indicated that **2** was a 6′,7′-dehydrogen derivative of **1**. This was confirmed by HMBC correlations from H-6′ (*δ*_H_ 5.82) to C-4′ (*δ*_C_ 33.3), C-5′ (*δ*_C_ 51.6) and C-8′ (*δ*_C_ 125.8), and from H-7′ (*δ*_H_ 6.37) to C-5′and C-14′ (*δ*_C_ 118.0). By detailed analysis of its ROESY (Fig. 2) spectrum, the relative configuration of **2** was also elucidated to be the same as that of **1**. Ultimately, the structure of **2** was determined and named salviwardin B.

**Fig. 3:**
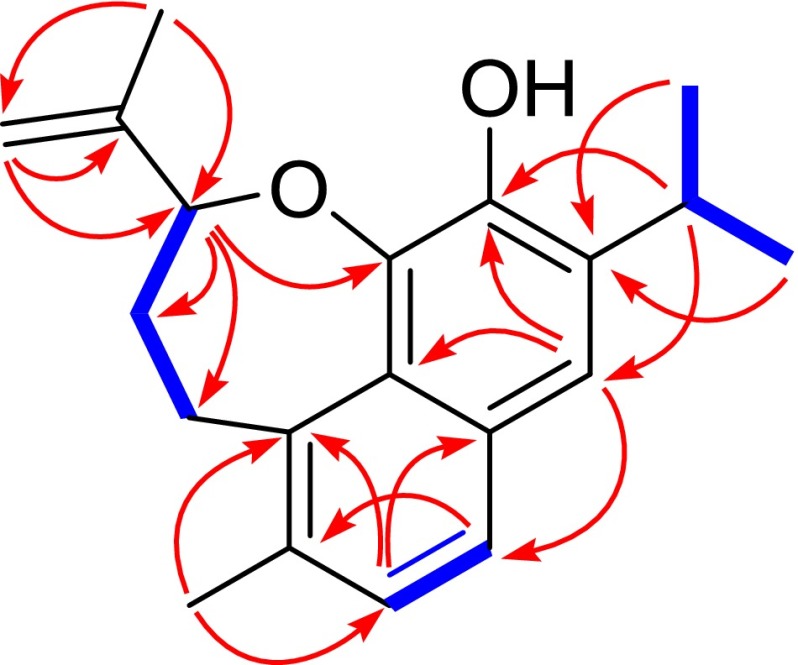
Key HMBC () and ^1^H-^1^H COSY () correlations of **3**

Salviwardin C (**3**) was assigned the molecular formula C_20_H_24_O_2_ by analysis of its ^13^C NMR and HREIMS (*m/z* 296.1775, [M]^+^). Comparing of NMR spectroscopic data of **3** (Table 3) with that of naphth-[1,8-bc]-oxocin-11-ol,2,3,4,5tetrhydro-2,2,6-trimethyl-10-(1-methlethyl) indicated that the two compounds are similar to each other. The difference lied in that the C-3, C-4, and Me-18 in the known compound were replaced by an oxygenated methine at *δ*_C_ 86.5 (C-3) and a part of double-bond (C-4, *δ*_C_ 145.5 and C-18, *δ*_C_ 111.0) in **3** [25]. The HMBC correlations from H-3 (*δ*_H_ 4.50) to C-1 (*δ*_C_ 26.0), C-2 (*δ*_C_ 33.6), C-4 (*δ*_C_ 145.5), C-11 (*δ*_C_ 141.0), C-18 (*δ*_C_ 111.0) and C-19 (*δ*_C_ 18.8), and from H-18 (*δ*_H_ 5.10 and 4.90) to C-3 (*δ*_C_ 86.5), C-4 (*δ*_C_ 145.5), C-19, together with the proton spin systems H-1/H-2/H-3, determined the structure of A ring of **3** as shown in Fig. 3. Other parts of **3** were identical to those of the known compound by detailed analysis of the ^1^H–^1^H COSY and HMBC correlations (Fig. 3). Therefore, the structure of **3** was determined and named salviwardin C.

**Table 3:** ^1^H and ^13^C NMR data for compound **3** in CDCl_3_

Position	*δ*_C_^a^	*δ*_H_ (*J* in Hz)^b^	Position	*δ*_C_^a^	*δ*_H_ (*J* in Hz)^b^
1	26.0, CH_2_	3.10, td (3.9,13.8)	11	141.0, C	
		3.41, dt (5.2,13.8)	12	143.7, C	
2	33.6, CH_2_	2.26, m	13	135.6, C	
3	86.5, CH	4.50, dd (7.2, 9.0)	14	119.6, CH	7.26, s
4	145.5, C		15	28.0, CH	3.34, sept (6.9)
5	131.6, C		16	23.0, CH_3_	1.29, d (6.9)
6	127.1, CH	7.02, d (8.3)	17	22.4, CH_3_	1.33, d (6.9)
7	125.9, CH	7.42, d (8.3)	18	111.0, CH_2_	4.90, s
8	128.2, C				5.10, s
9	126.7, C		19	18.8, CH_3_	1.85, s
10	132.3, C		20	20.3, CH_3_	2.42, s

^a^Recorded at 150 MHz

^b^Recorded at 600 MHz

All isolates were tested for their in vitro inhibitory activities against HL-60, SMMC-7721, A549, MCF-7, and SW480 human tumor cell lines using the MTT method described previously [26]. The results indicated that all the compounds were inactive with IC_50_ > 30 μM.

## Experiment Section

### General Experimental Procedures

Optical rotations were obtained with a Jasco P-1020 polarimeter. UV spectra were measured on Shimadzu UV-2401A spectraphotometer. IR spectra were detected on a Bruker Tensor-27 infrared spectrophotometer with KBr pellets. 1D and 2D NMR spectra were recorded on Bruker AV-400, and Avance III-600 MHz spectrometers with TMS as the internal standard. Chemical shifts (*δ*) were expressed in ppm with reference to the solvent signals. HRESIMS analysis and HREIMS were determined on API QSTAR time-of-flight spectrometer and on Waters Auto spec Premier P776 mass spectrometer. Semi-preparative HPLC was performed on an Agilent 1100 liquid chromatography with a Zorbax SB-C18 (9.4 mm × 25 cm) column. Column chromatography was performed on Sephadex LH-20 (GE Healthcare), Silica gel (100–200 and 200–300 mesh, Qingdao Marine Chemical Co., Ltd., Qingdao, China), and Amphichroic RP-18 gel (40–63 μm, Merck, Darmstadt, Germany) and MCI gel (75–150 μm, Mitsubishi Chemical Corporation, Tokyo, Japan). Fractions were monitored by TLC and spots were visualized by heating silica gel plates sprayed with 10 % H_2_SO_4_ in EtOH.

### Plant Material

The roots parts of *S. wardii* were collected in Zuogong prefecture Tibet, China, in July 2011. The plant was identified by Dr. Yu-Kun Wei, Shanghai Chenshan Plant Science Research Center, Chinese Academy of Sciences. A voucher specimen was deposited in Kunming Institute of Botany,Chinese Academy of Sciences with identification number 20110712.

### Extraction and Isolation

The roots parts of the air-dried *S. wardii* (33 kg) were powdered and percolated with acetone at room temperature and filtered. The filtrate was evaporated in vacuo to be concentrated. The crude extract (1.6 kg) was subjected to silica gel column chromatography eluted with CHCl_3_, EtoAc and MeOH, respectively. The fraction CHCl_3_ with petroleum-CHCl_3_-EtoAc gradient (50:1:1, 20:1:1, 10:1:1, and 5:1:1) to produce seven fractions, A–G. Fraction B (223 g) was separated over a MCI gel column (MeOH-H_2_O from 70:30 to 100:0) to obtain eight fractions (Fr. B1–B8). Fr. B1 (12 g) was isolated over an RP-18 gel column (MeOH–H_2_O from 85:15 to 100:0) to obtain seven fractions (Fr. B2a–B2g). Fr. B2a (2 g) was separated on a silica gel column, eluted with petroleum ether-acetone (from 100:1 to 50:1), to yield six fractions (B2a1–B2a6). Fr. B2a2 was purified by repeated silica gel columns and semipreparative HPLC (RP-18, 98 % MeCN-H_2_O) and TLC to afford **1** (80 mg), **2** (13 mg). Fr. B3 (27 g) was separated over a MCI-gel column (MeOH-H_2_O from 85:15 to 100:0) to obtain six fractions (Fr. B3a–B3f). Fr. B3e (5 g) was then chromatographed on a silica gel column, eluted with petroleum ether-acetone (from 50:1 to 5:1), to yield eight fractions (Fr. B3e1–B3e8). Subfraction B3e1 (110 mg) was chromatographed by semipreparative HPLC (89 % MeOH-H_2_O) to afford two fractions (Fr. B3e1a–Fr. B3e1b). Fr. B3e1a (15 mg) was chromatographed by semipreparative HPLC (92 % MeCN-H_2_O) to afford **3** (10 mg). Subfraction B3e1 (1.7 g) was chromatographed by semipreparative HPLC (83 % MeOH-H_2_O) to afford two fractions (Fr. B3e2a–Fr. B3e1f). Fr. B3e1a (15 mg) was chromatographed by semipreparative HPLC (92 % MeCN-H_2_O) and chromatographed on a silica gel column, eluted with petroleum ether-acetone (100:1) to afford prionitin (**4**, 100 mg). Fr. B3f (3.3 g) was then chromatographed on a silica gel column, eluted with petroleum ether-acetone (from 50:1 to 5:1), to yield eight fractions (Fr. B3f1–B3f7). Fr. B3f1 (726 mg) was then chromatographed on a silica gel column, eluted with petroleum ether-acetone (50:1), to yield eight fractions (Fr. B3f1a–B3f1f). Fr. Bf1a, Fr. Bf1c, and Fr. Bf1f were purified by semipreparative TLC and chromatographed by semipreparative HPLC (92 % MeCN-H_2_O) to yield sahandol (**5**, 49 mg), salvilenone (**6**, 10 mg), microstegiol (**7**, 6 mg), and ferruginol (**8**, 120 mg).

### Salviwardin A (**1**)

Orange powder; $[\alpha]_{\text{D}}^{16}$ + 325 (*c* 0.17, CDCl_3_); UV (CDCl_3_) *λ*_max_ (log *ε*) 374.5 (3.68) nm; IR (KBr) *ν*_max_ 3441, 2939, 1624, 1476, 1417, 1364, 1296, 1134, 1121, 1011 cm^−1^; ^1^H and ^13^C NMR data, see Tables 1 and 2; positive ESIMS *m/z* 599 [M + H]^+^; positive HREIMS *m/z* 598.4029 (calcd for C_40_H_54_O_4_ [M]^+^, 598.4022).

### Salviwardin B (**2**)

Orange powder; $[\alpha ]_{\text{D}}^{20}$ + 184 (*c* 0.19, MeOH); UV (MeOH) *λ*_max_ (log *ε*) 476 (3.25) nm; IR (KBr) *ν*_max_ 3442, 2927, 2870, 1626, 1579, 1467, 1394, 1291, 1172, 1088, 1008 cm^−1^; ^1^H and ^13^C NMR data, see Tables 1 and 2; positive ESIMS *m/z* 619 [M + Na]^+^; positive HRESIMS *m/z* 597.3941 (calcd for C_40_H_53_O_4_ [M + H]^+^, 597.3944).

### Salviwardin C (**3**)

Colorless oil; $[\alpha ]_{\text{D}}^{26}$ −2 (*c* 0.26, CDCl_3_); UV (CDCl_3_) *λ*_max_ (log *ε*) 336.5 (3.35) nm; IR (KBr) *ν*_max_ 3430, 2927, 2871, 1722, 1633, 1423, 1330, 1271, 1172, 1023 cm^−1^; ^1^H and ^13^C NMR data see Table 3; positive EIMS *m/z* 296 ([M]^+^, 77), 268 (26), 267 (94), 265 (100), 255 (18), 241 (35), 213 (54); positive HREIMS *m/z* 296.1775 (calcd for C_20_H_24_O_2_ [M]^+^, 296.1776).

### Cytotoxicity Assays

The following human tumor cell lines were used: HL-60, SMMC-7721, A-549, MCF-7, and SW-480, which were obtained from ATCC (Manassas, VA, USA). All cells were cultured in RPMI-1640 or DMEM medium (Hyclone, Logan, UT, USA), supplemented with 10 % fetal bovine serum (FBS, Hyclone) at 37 °C in a humidified atmosphere with 5 % CO_2_. Cell viability was assessed by conducting colorimetric measurements of the amount of insoluble formazan formed in living cells based on the reduction of 3-(4,5-dimethylthiazol-2-yl)-2,5-diphenyltetrazolium bromide (MTT) (Sigma, St. Louis, MO, USA). Briefly, 100 μL of adherent cells was seeded into each well of a 96-well cell culture plate and allowed to adhere for 12 h before test compound addition, while suspended cells were seeded just before test compound addition, both with an initial density of 1 × 10^5^ cells/mL in 100 μL of medium. Each tumor cell line was exposed to the test compound at various concentrations in triplicate for 48 h, with *cis*-platin and paclitaxel (Sigma) as positive control. After the incubation, MTT (100 μg) was added to each well, and the incubation continued for 4 h at 37 °C. The cells were lysed with 100 μL of 20 % SDS −50 % DMF after removal of 100 μL of medium. The optical density of the lysate was measured at 595 nm in a 96-well microtiter plate reader (Bio-Rad 680). The IC50 value of each compound was calculated by Reed and Muench’s method [25].

## Electronic supplementary material

Below is the link to the electronic supplementary material.

Supplementary material 1 (PDF 1512 kb)
