# Erratum to: New Dimeric and *seco*-Abietane Diterpenoids from *Salvia wardii*

**DOI:** 10.1007/s13659-015-0061-7

**Published:** 2015-05-30

**Authors:** Qiu-Li Xiao, Fan Xia, Xing-Wei Yang, Yang Liao, Li-Xin Yang, Yu-Kun Wei, Xian Li, Gang Xu

**Affiliations:** School of Pharmaceutical Science and Yunnan Key Laboratory of Pharmacology of Natural Products, Kunming Medical University, Kunming, 650500 Yunnan People’s Republic of China; State Key Laboratory of Phytochemistry and Plant Resources in West China, Kunming Institute of Botany, Chinese Academy of Sciences, Kunming, 650201 People’s Republic of China; Shanghai Chenshan Plant Science Research Center, Chinese Academy of Sciences, Beijing, People’s Republic of China

## Erratum to: Nat. Prod. Bioprospect. (2015) 5:77–82 DOI 10.1007/s13659-015-0054-6

In the HTML version of the original publication, the graphical abstract was inadvertently omitted. The graphical abstract is given in this erratum.



