# Supplementary material for: New Dimeric and seco-Abietane Diterpenoids from Salvia wardii
Source: Nat Prod Bioprospect. 2015 Apr 8;5(2):77–82. doi: 10.1007/s13659-015-0054-6 (PMC4402580; doi:10.1007/s13659-015-0054-6)
Supplement: Supplementary file 1 — Supplementary material 1 (PDF 1512 kb) [file 13659_2015_54_MOESM1_ESM.pdf]

Electronic Supplementary Material

New Dimeric and *seco*-Abietane Diterpenoids from *Salvia wardii*

Qiu-Li Xiao, Fan Xia, Xing-Wei Yang, Yang Liao, Li-Xin Yang, Yu-Kun Wei, Xian Li, and Gang Xu

Figures S1–S30: The original NMR and MS spectra of the new compounds.

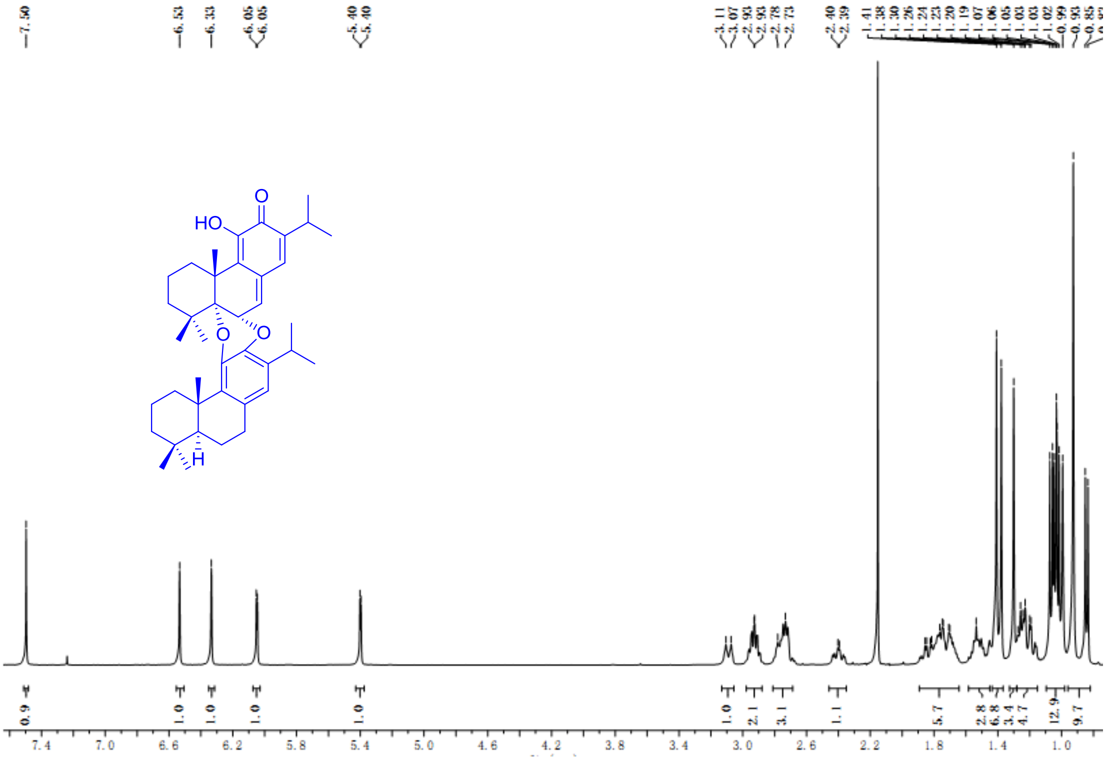

Figure S1. ^{1}H NMR spectrum of salviwardin A (**1**) in $CDCl_3$ (400 MHz).

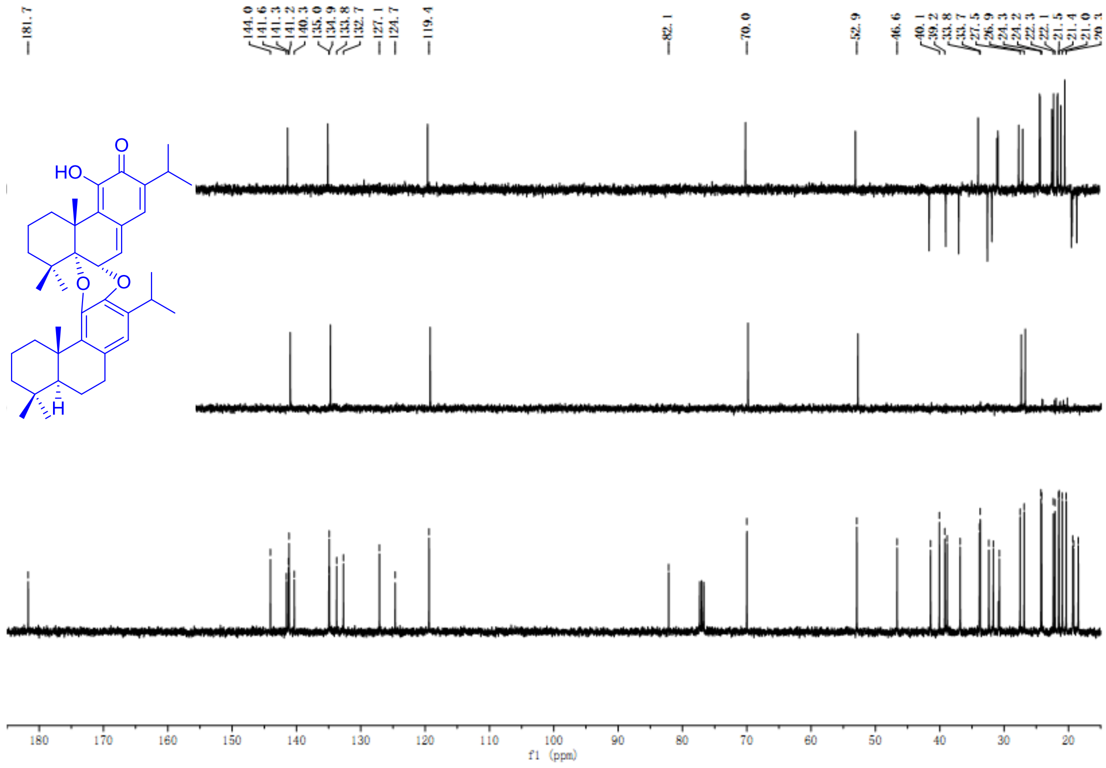

Figure S2. ^{13}C and DEPT NMR spectra of salviwardin A (**1**) in $CDCl_3$ (400 MHz).

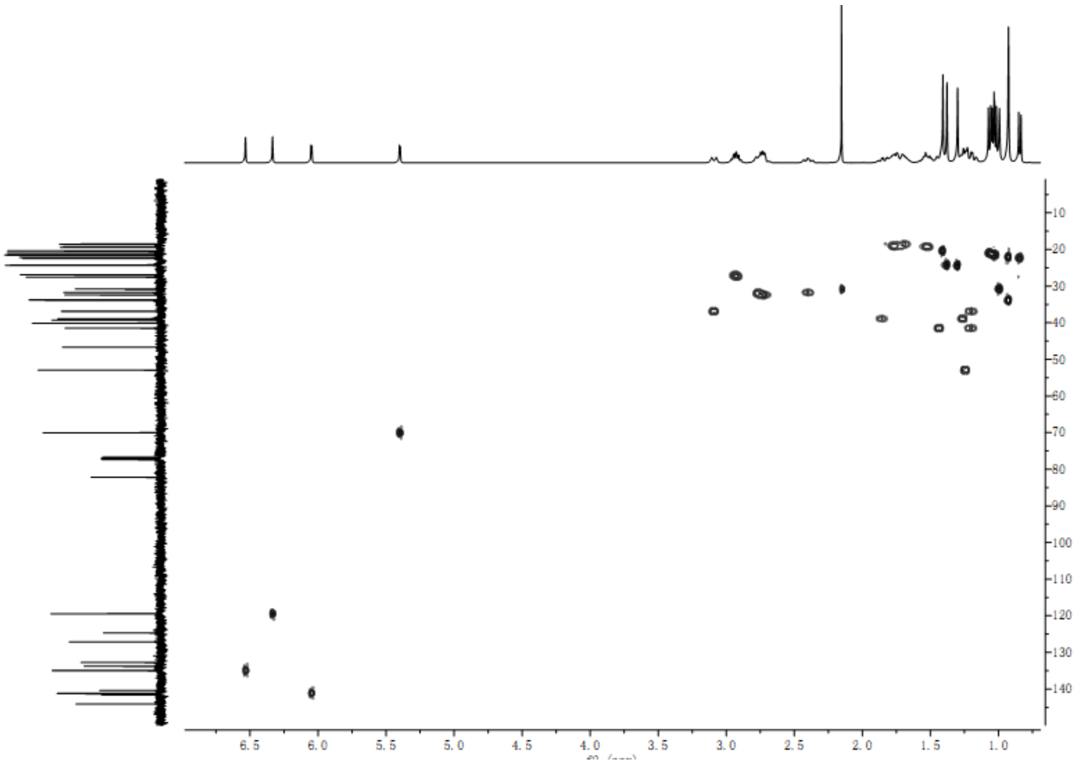

Figure S3. HSQC spectrum of salviwardin A (**1**).

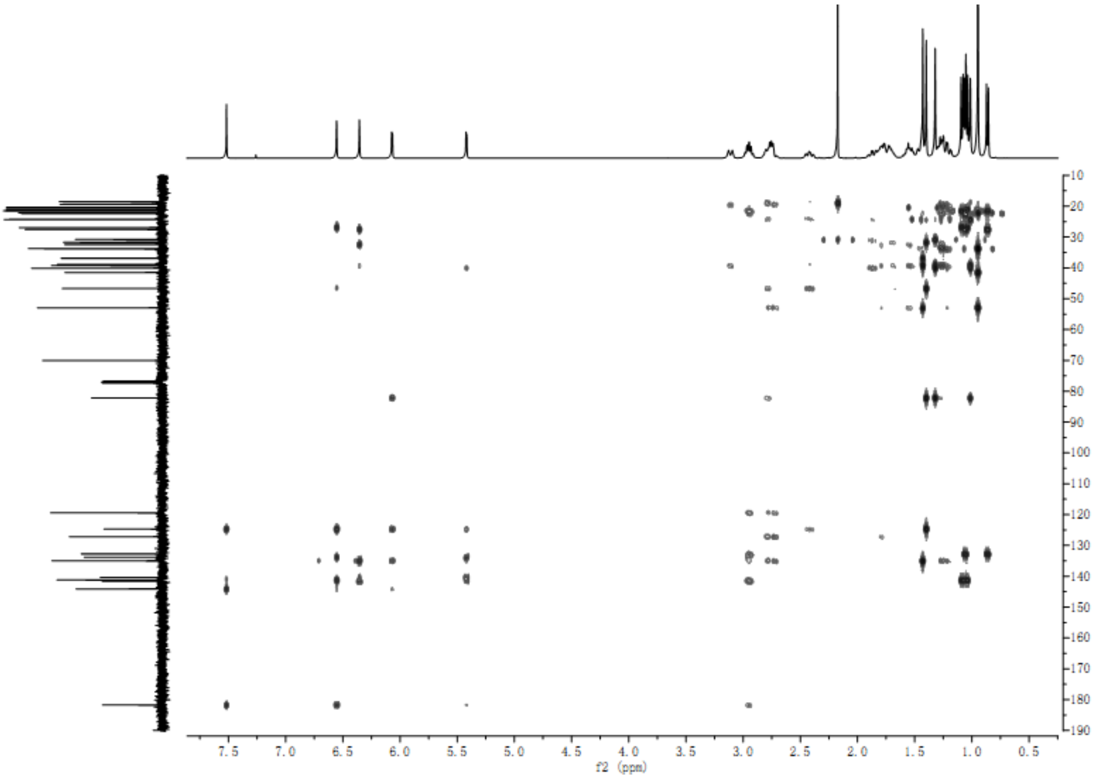

Figure S4. HMBC spectrum of salviwardin A (**1**).

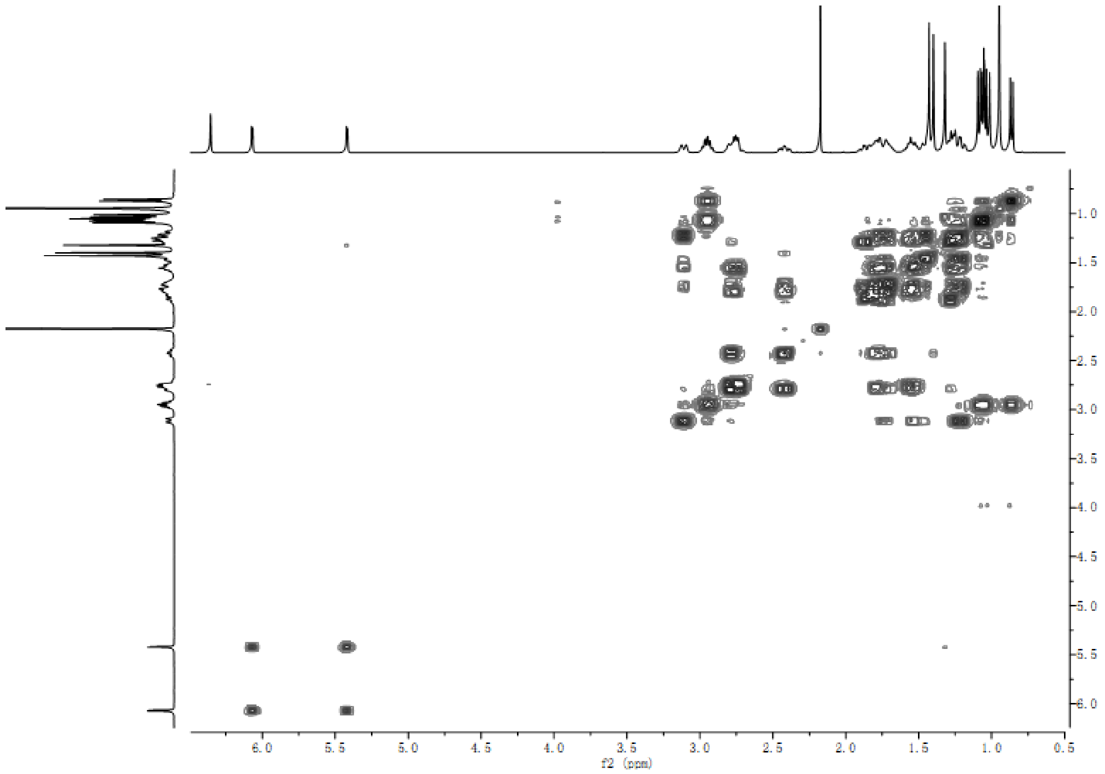

Figure S5. COSY spectrum of salviwardin A (**1**).

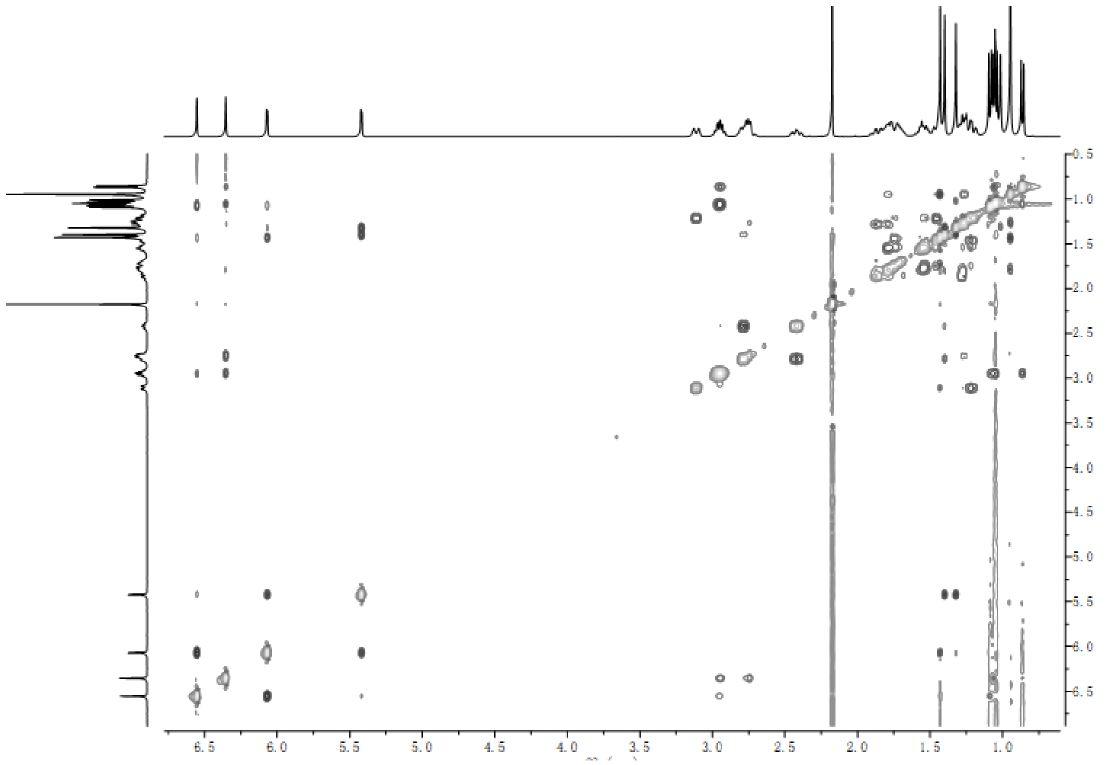

Figure S6. ROESY spectrum of salviwardin A (**1**).

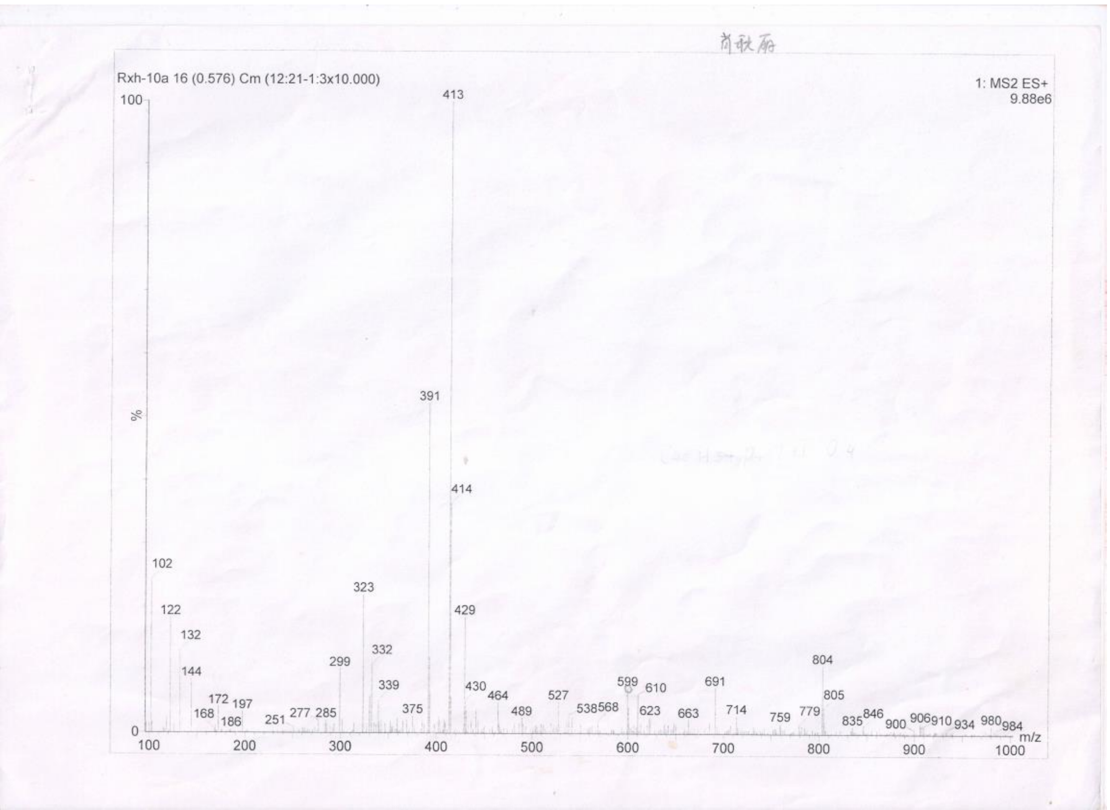

Figure S7. ESI-MS spectrum of salviwardin A (**1**).

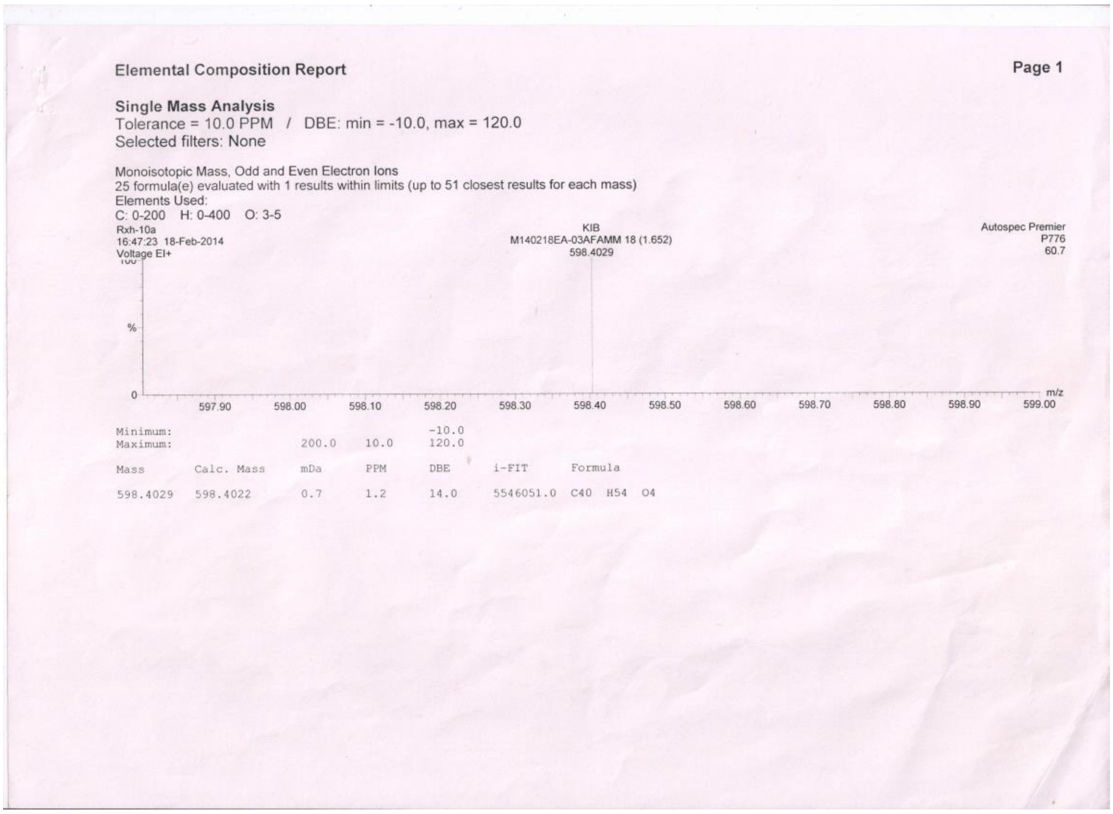

Elemental Composition Report **Page 1**

Single Mass Analysis
Tolerance = 10.0 PPM / DBE: min = -10.0, max = 120.0
Selected filters: None

Monoisotopic Mass, Odd and Even Electron Ions
25 formula(e) evaluated with 1 results within limits (up to 51 closest results for each mass)
Elements Used:
C: 0-200 H: 0-400 O: 3-5

Rxh-10a
16:47:23 18-Feb-2014
Voltage EI+

KIB
M140218EA-03AFAMM 18 (1.652)
598.4029

Autospec Premier
P776
60.7

Minimum:				-10.0		
Maximum:		200.0	10.0	120.0		
Mass	Calc. Mass	mDa	PPM	DBE	i-FIT	Formula
598.4029	598.4022	0.7	1.2	14.0	5546051.0	C40 H54 O4

Figure S8. HREI MS spectrum of salviward A (**1**).

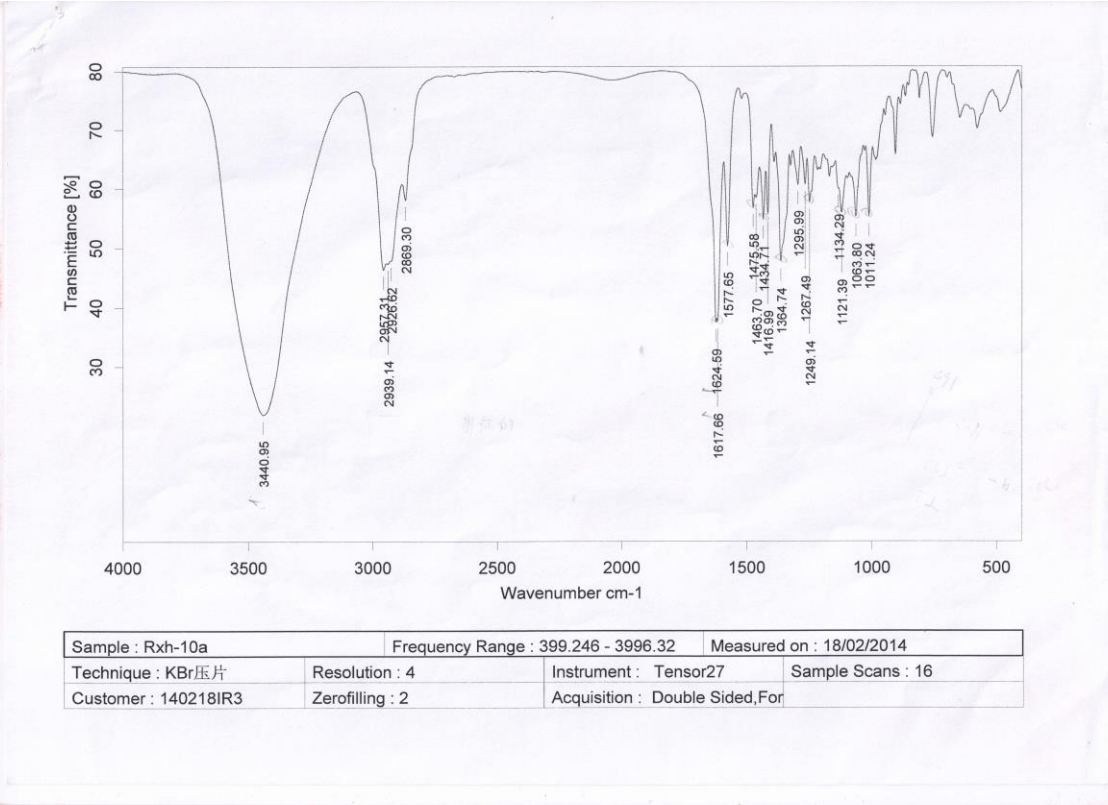

Figure S9. IR (KBr disk) spectrum of salviwardin A (**1**).

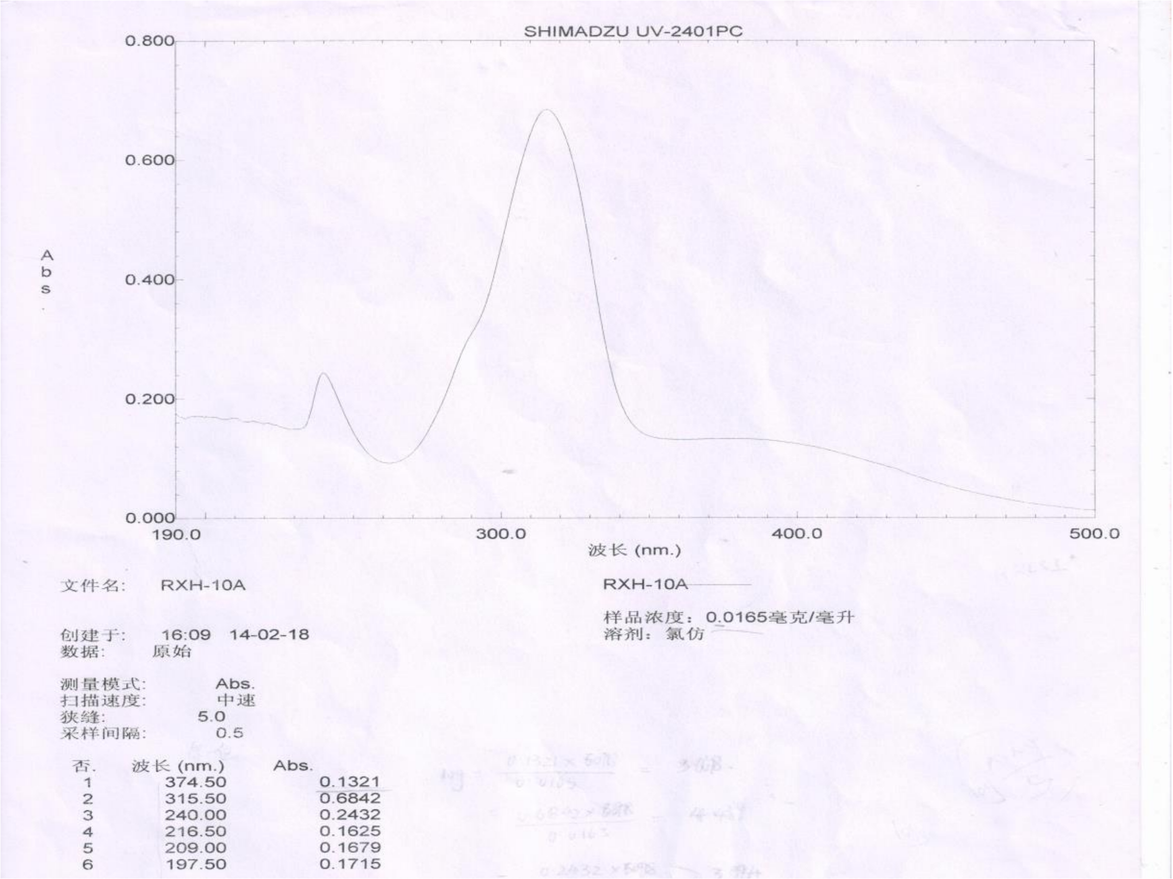

Figure S10. UV spectrum of salviwardin A (**1**).

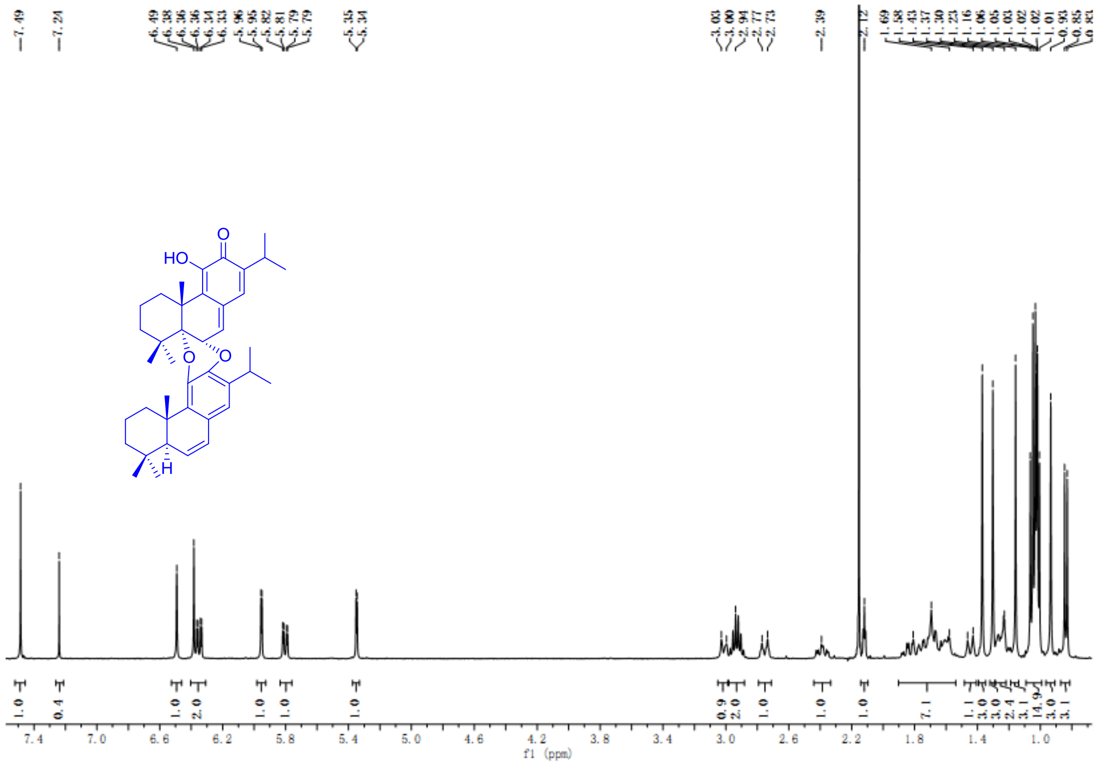

Figure S11. ^{1}H NMR spectrum of salviwardin B (**2**) in $CDCl_3$ (400 MHz).

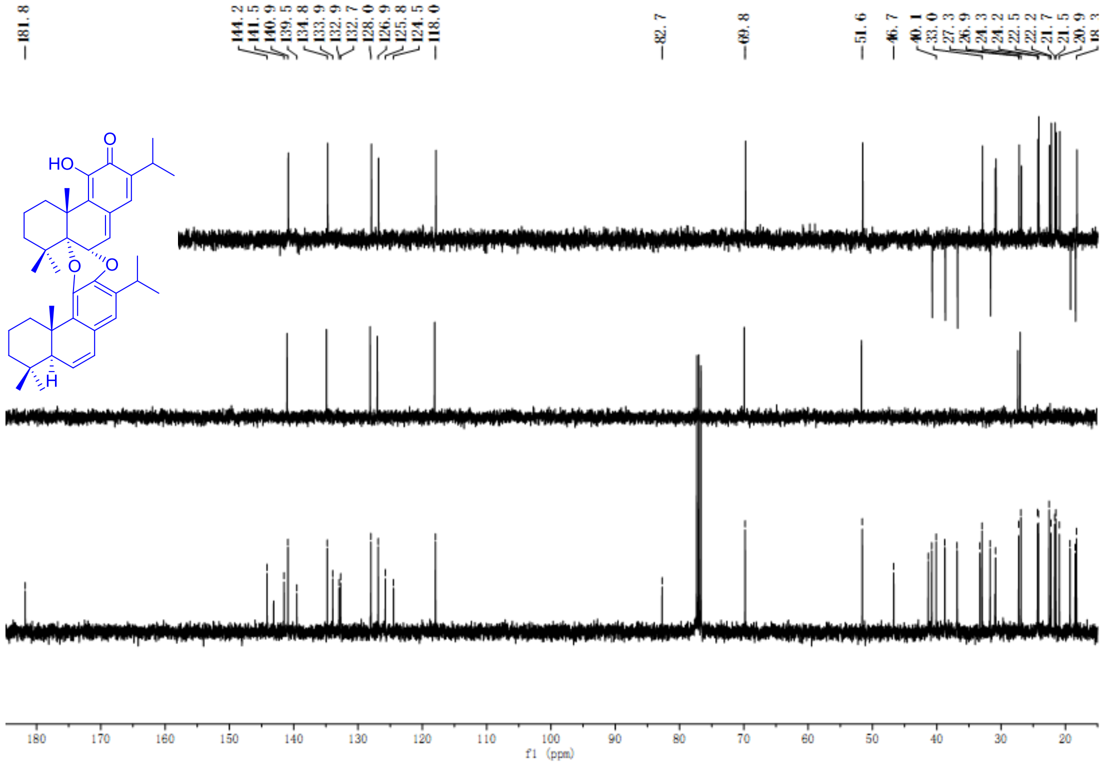

Figure S12. ^{13}C and DEPT NMR spectra of salviwardin B (**2**) in $CDCl_3$ (400 MHz).

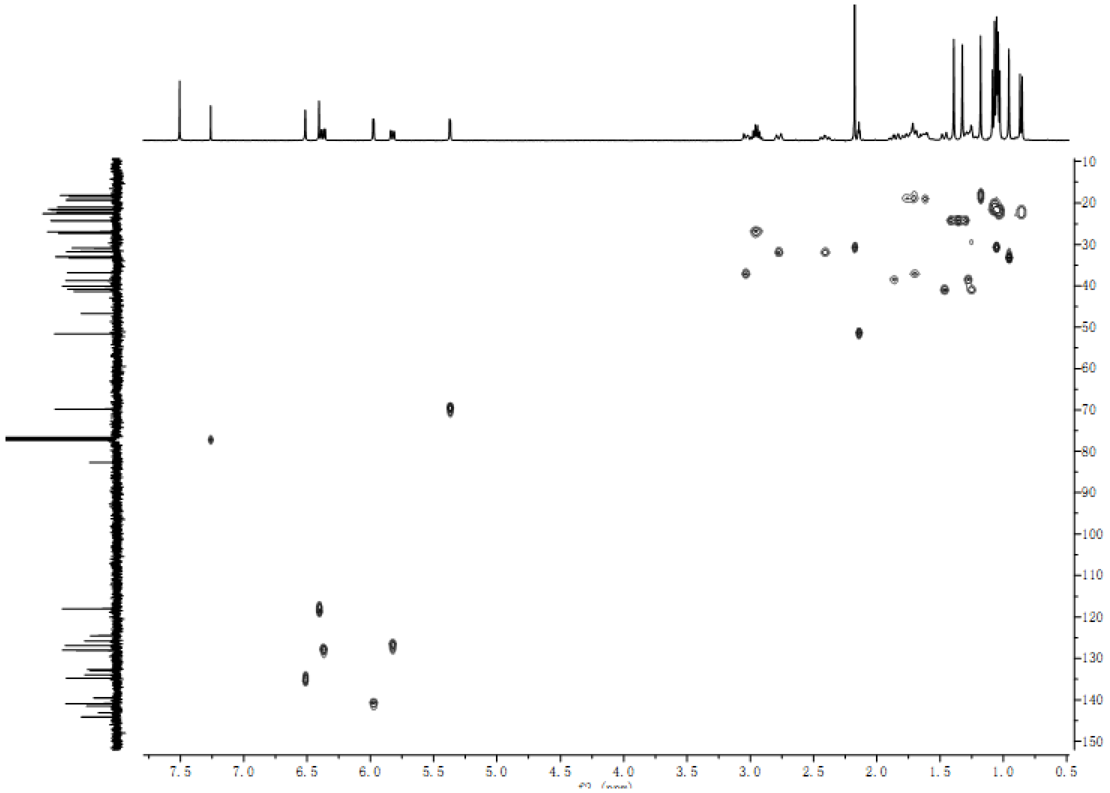

Figure S13. HSQC spectrum of salviwardin B (**2**).

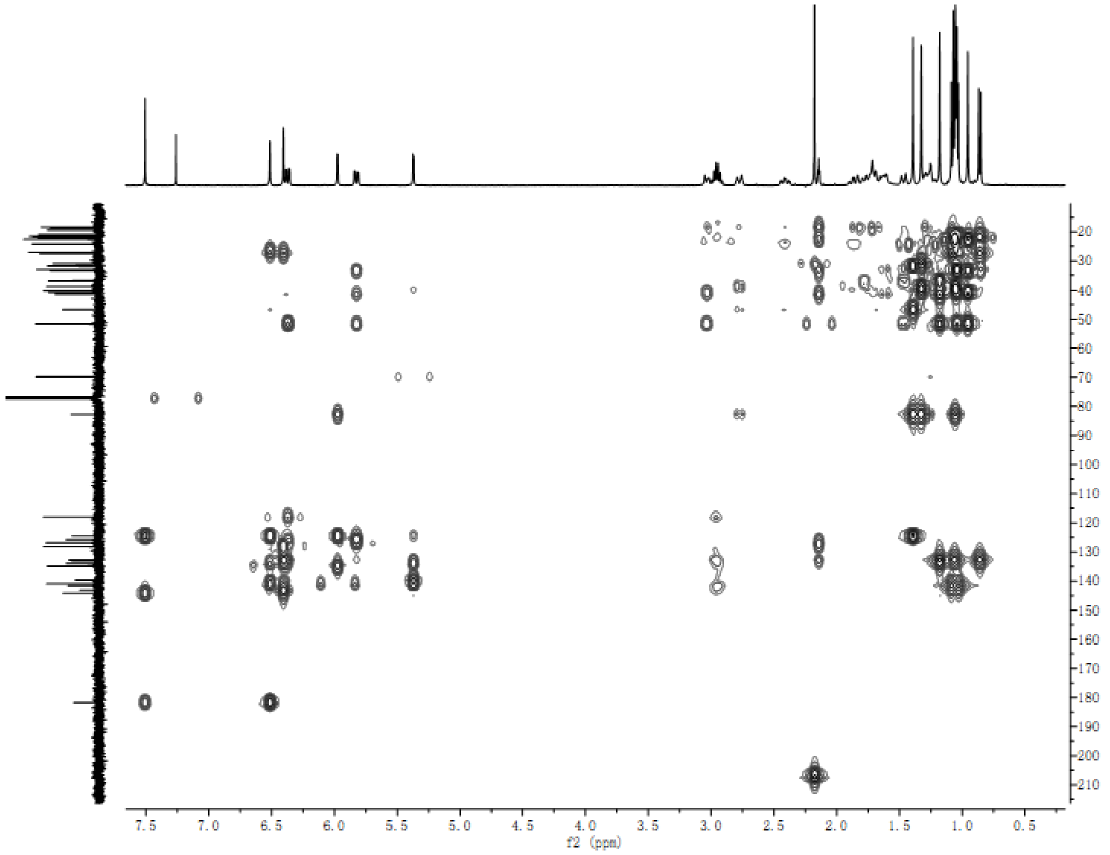

Figure S14. HMBC spectrum of salviwardin B (**2**).

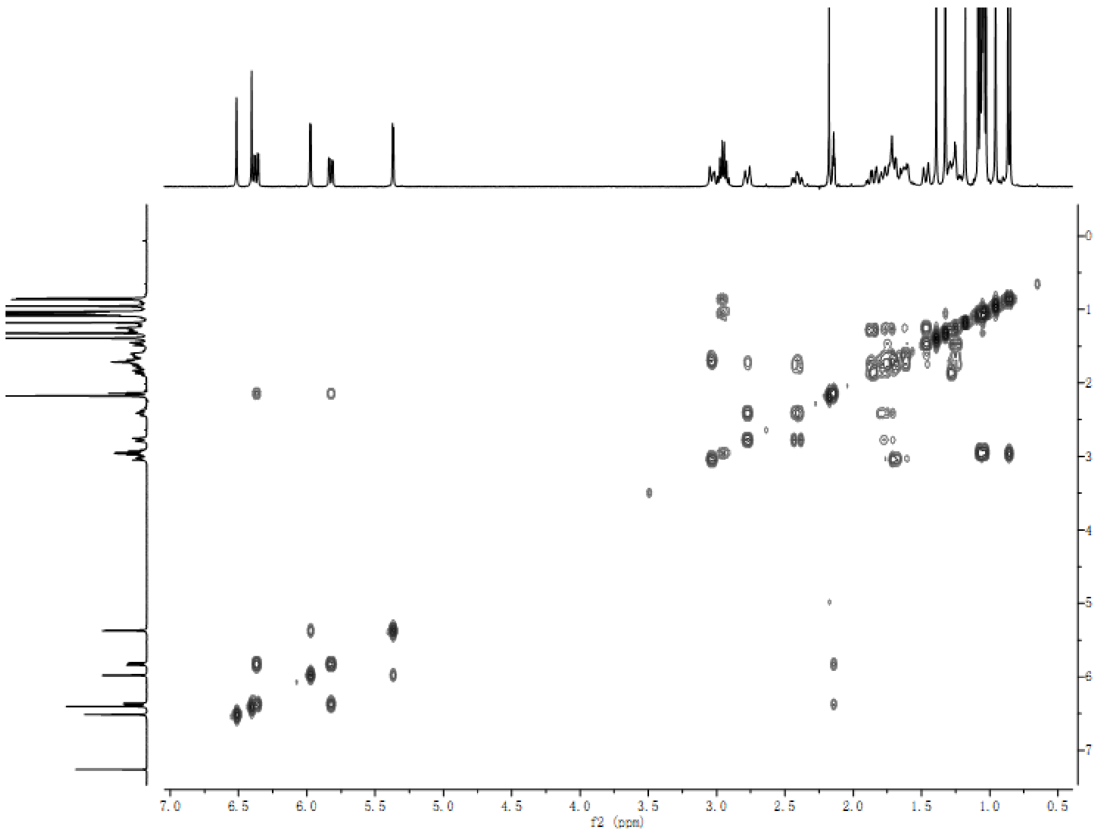

Figure S15. COSY spectrum of salviwardin B (**2**).

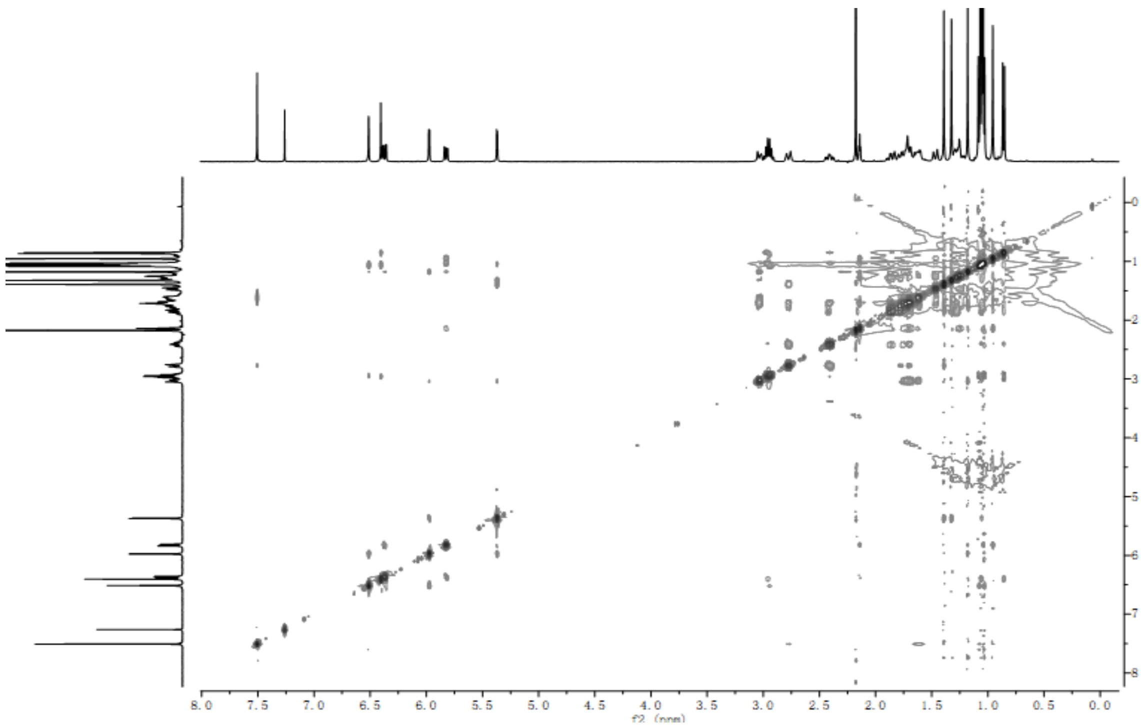

Figure S16. ROESY spectrum of salviwardin B (**2**).

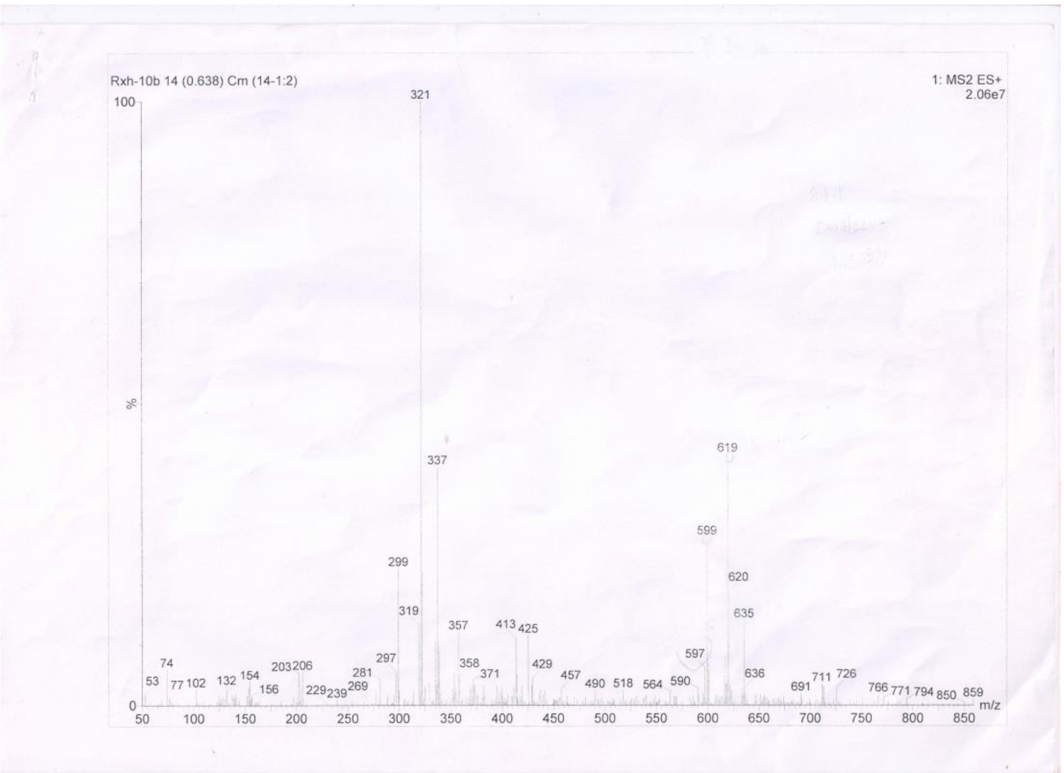

Figure S17. ESI-MS spectrum of salviwardin B (**2**).

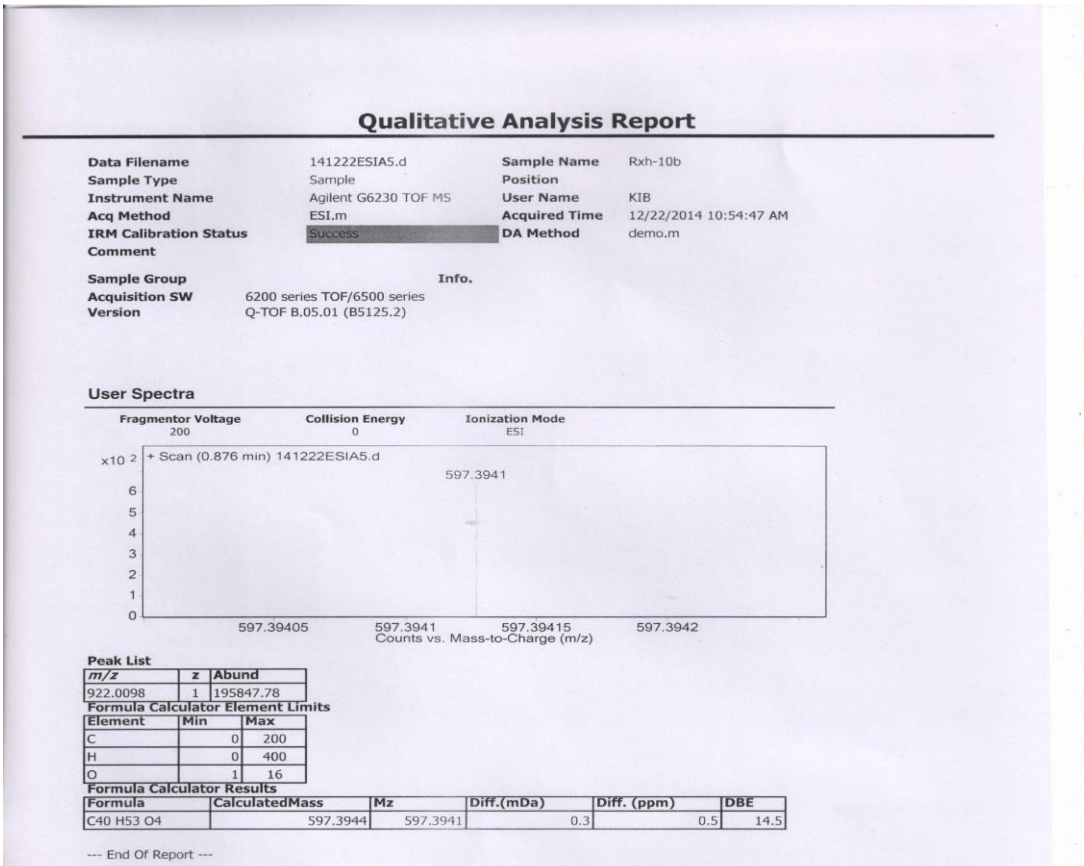

Qualitative Analysis Report

**Data Filename**	141222ESIA5.d	**Sample Name**	Rxh-10b
**Sample Type**	Sample	**Position**	
**Instrument Name**	Agilent G6230 TOF MS	**User Name**	KIB
**Acq Method**	ESI.m	**Acquired Time**	12/22/2014 10:54:47 AM
**IRM Calibration Status**	Success	**DA Method**	demo.m
**Comment**			

**Sample Group**		**Info.**
**Acquisition SW Version**	6200 series TOF/6500 series Q-TOF B.05.01 (B5125.2)	

User Spectra

Fragmentor Voltage	Collision Energy	Ionization Mode
200	0	ESI

Peak List

m/z	z	Abund
922.0098	1	195847.78

Formula Calculator Element Limits

Element	Min	Max
C	0	200
H	0	400
O	1	16

Formula Calculator Results

Formula	CalculatedMass	Mz	Diff.(mDa)	Diff. (ppm)	DBE
C40 H53 O4	597.3944	597.3941	0.3	0.5	14.5

--- End Of Report ---

Figure S18. HREI-MS spectrum of salviwardin B (**2**).

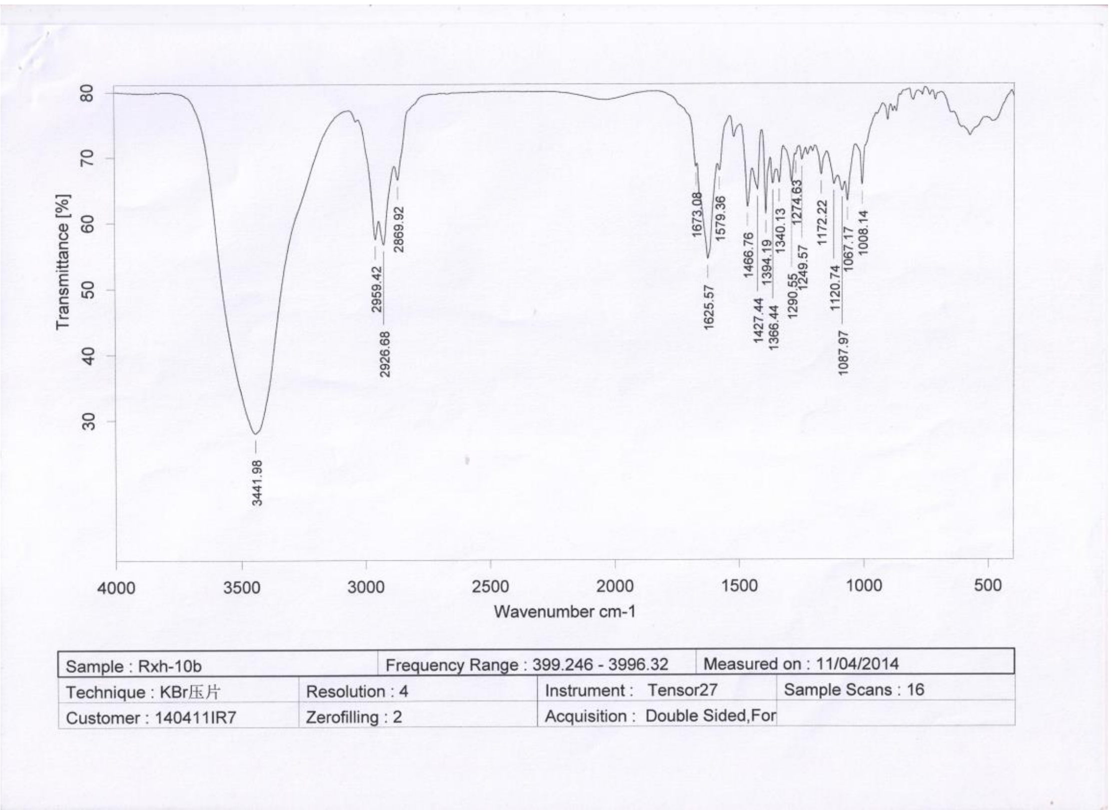

Figure S19. IR (KBr disk) spectrum of salviwardin B (**2**).

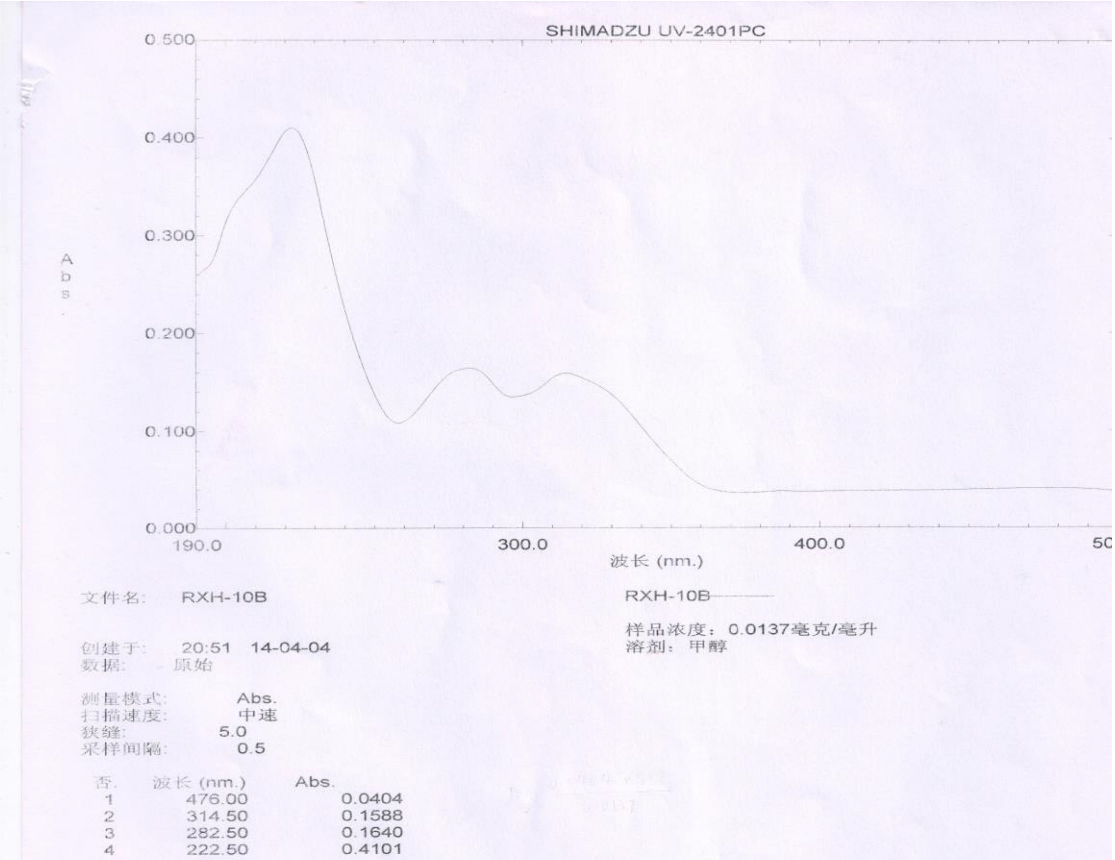

Figure S20. UV spectrum of salviwardin B (**2**).

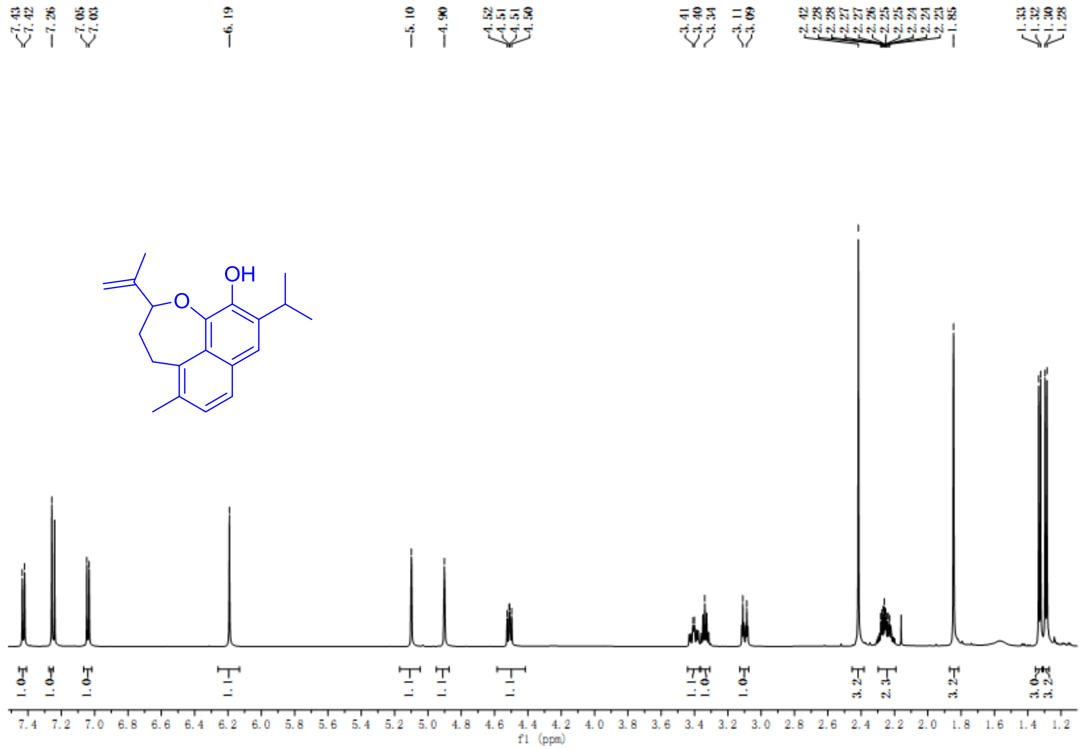

Figure S21. ^{1}H NMR spectrum of salviwardin C (**3**) in $CDCl_3$ (600 MHz).

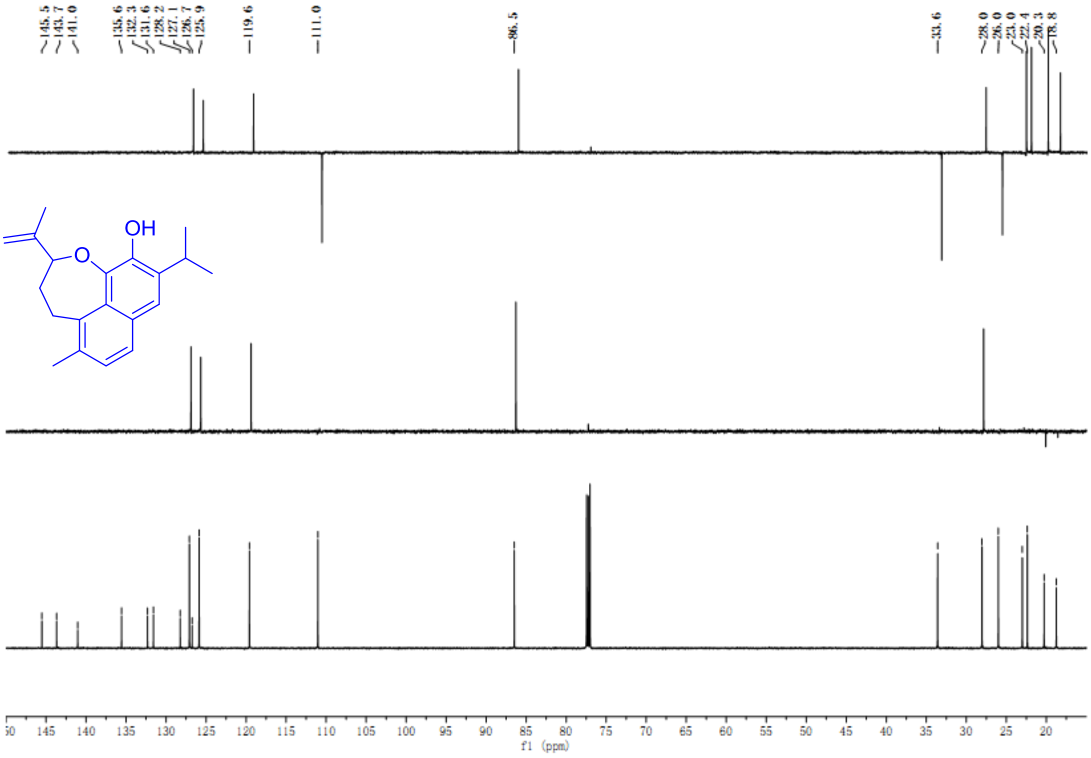

Figure S22. ^{13}C and DEPT NMR spectra of salviwardin C (**3**) in $CDCl_3$ (600 MHz).

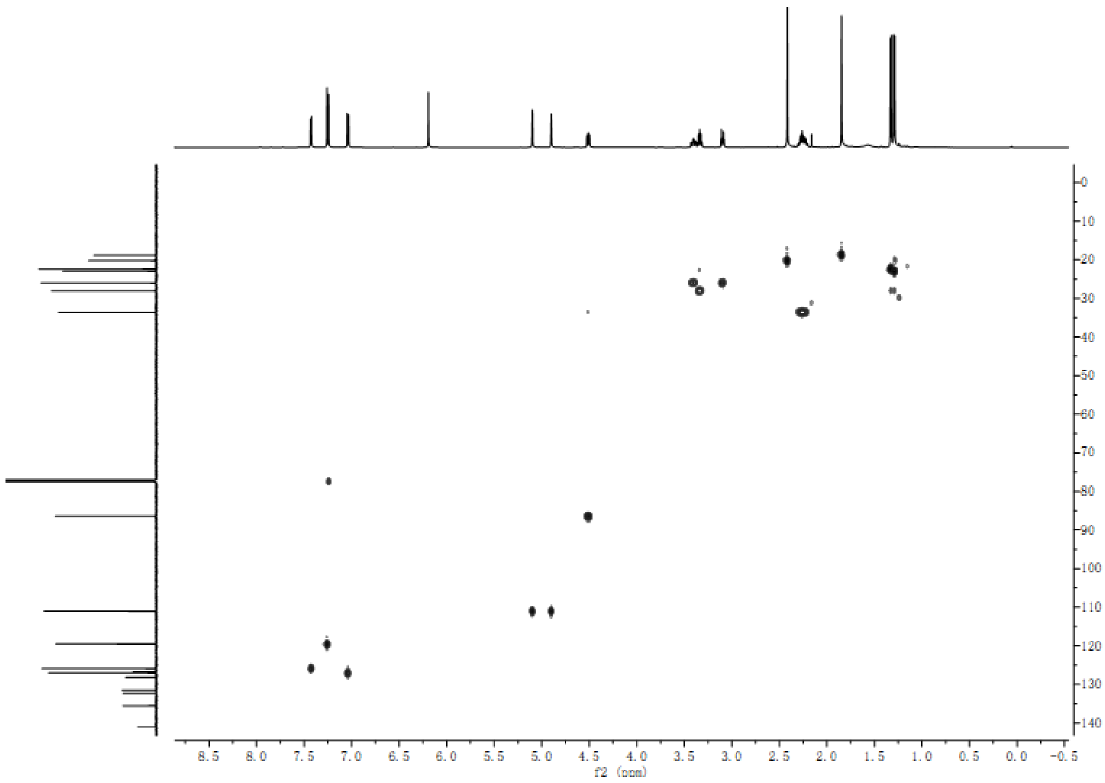

Figure S23. HSQC spectrum of salviwardin C (**3**).

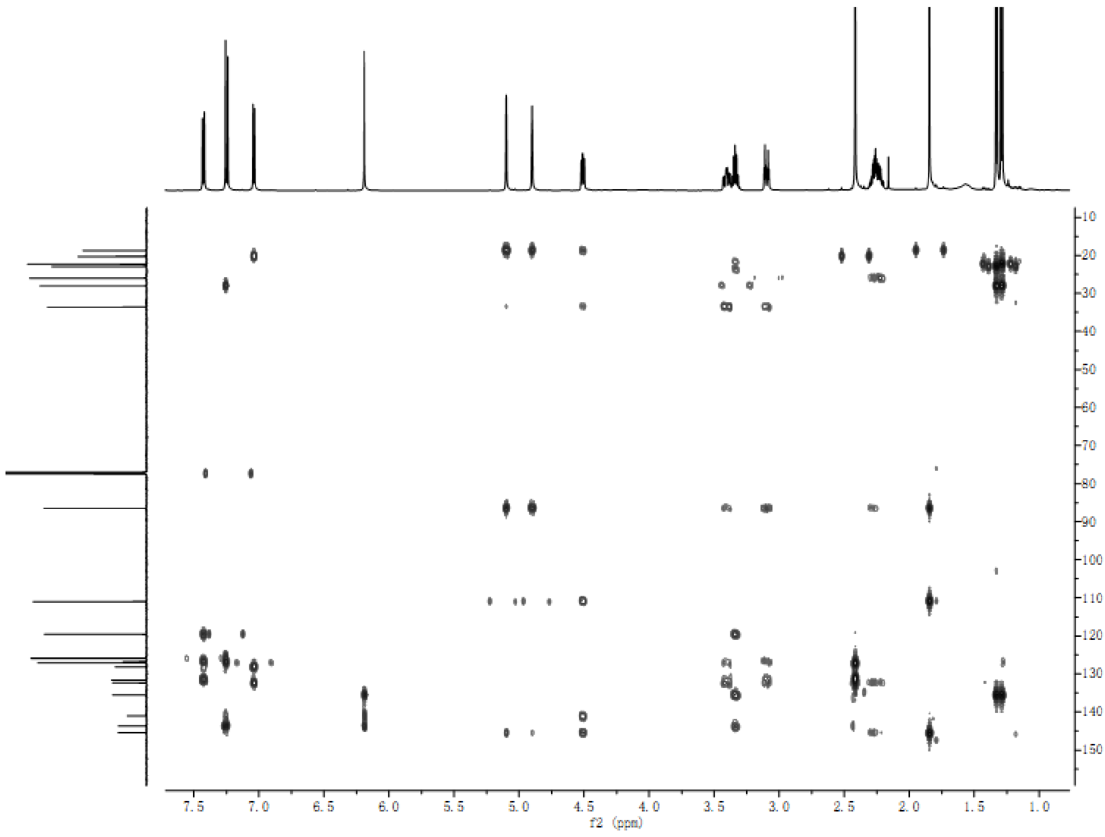

Figure S24. HMBC spectrum of salviwardin C (**3**).

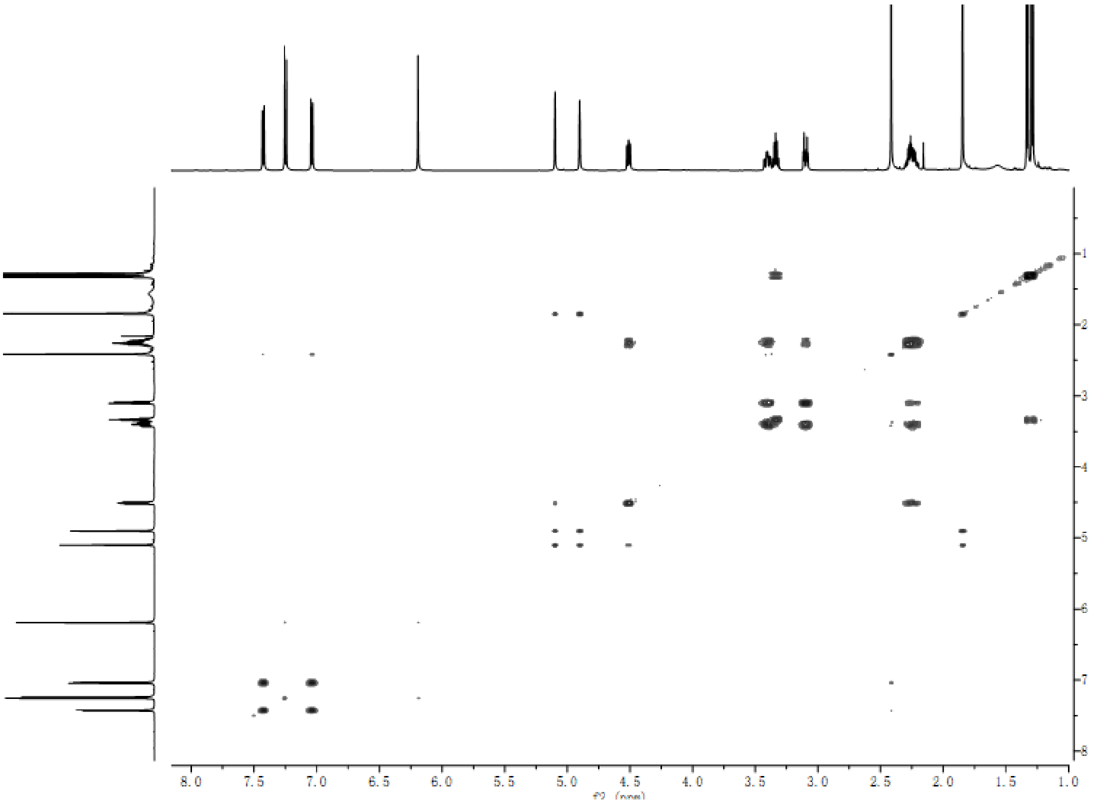

Figure S25. COSY spectrum of salviwardin C (**3**).

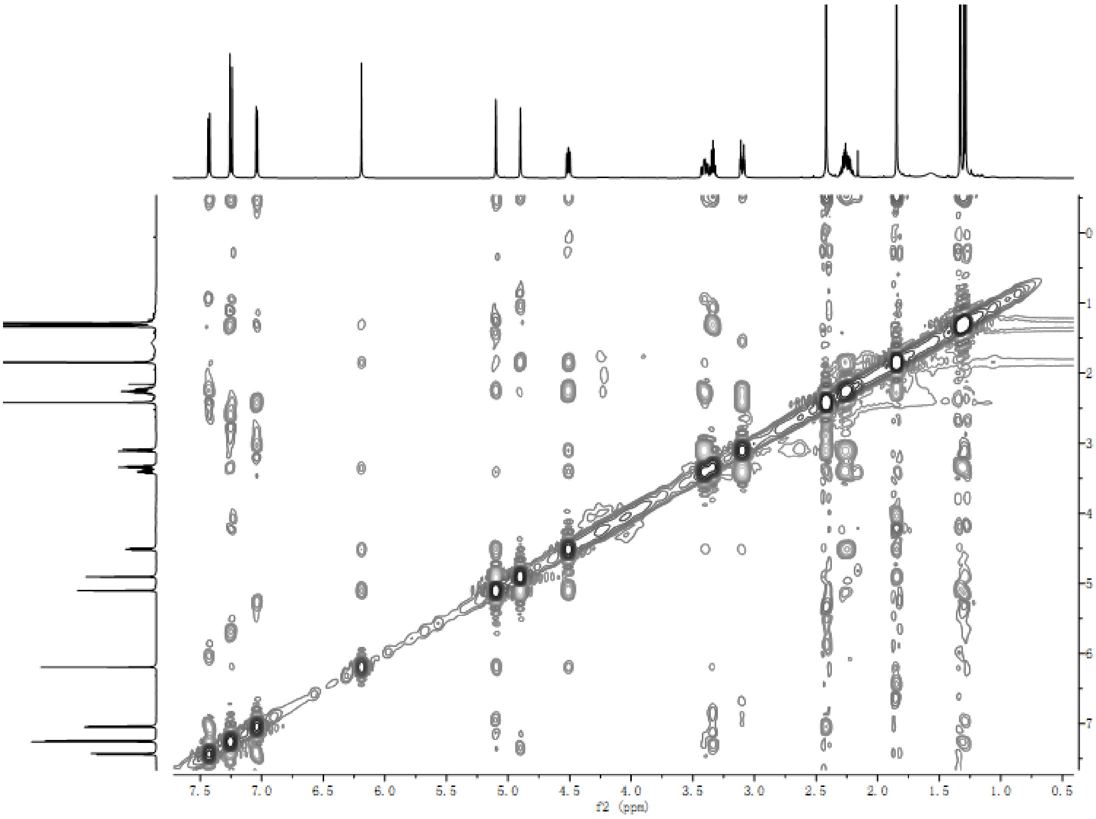

Figure S26. ROESY spectrum of salviwardin C (**3**).

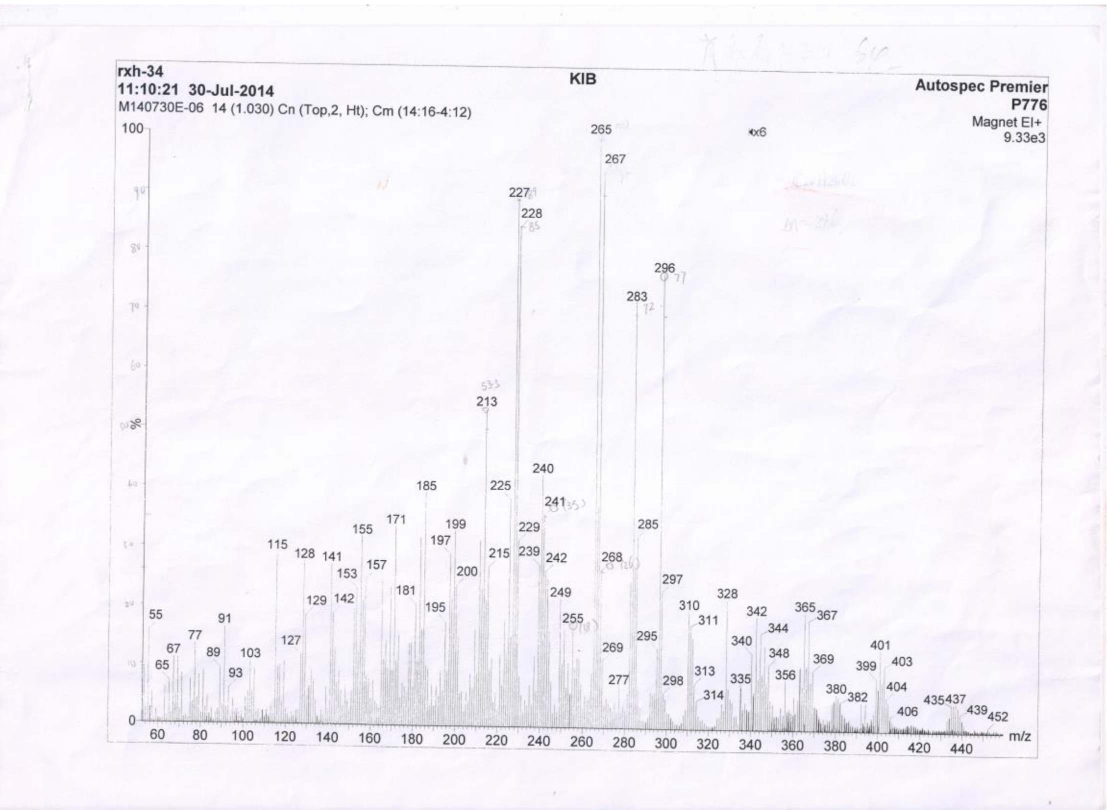

Figure S27. EI-MS spectrum of salviwardin C (**3**).

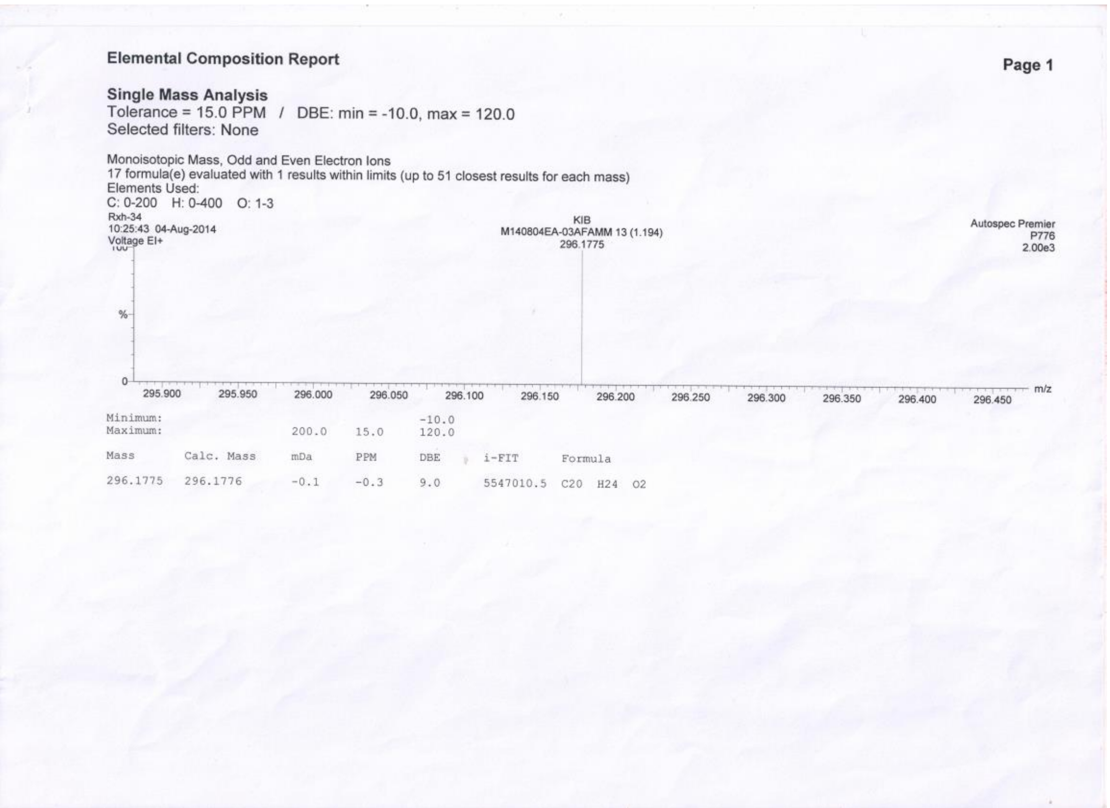

Elemental Composition Report

Page 1

Single Mass Analysis
Tolerance = 15.0 PPM / DBE: min = -10.0, max = 120.0
Selected filters: None

Monoisotopic Mass, Odd and Even Electron Ions
17 formula(e) evaluated with 1 results within limits (up to 51 closest results for each mass)
Elements Used:
C: 0-200 H: 0-400 O: 1-3

Minimum:				-10.0		
Maximum:		200.0	15.0	120.0		
Mass	Calc. Mass	mDa	PPM	DBE	i-FIT	Formula
296.1775	296.1776	-0.1	-0.3	9.0	5547010.5	C20 H24 O2

Figure S28. HREI-MS spectrum of salviwardin C (**3**).

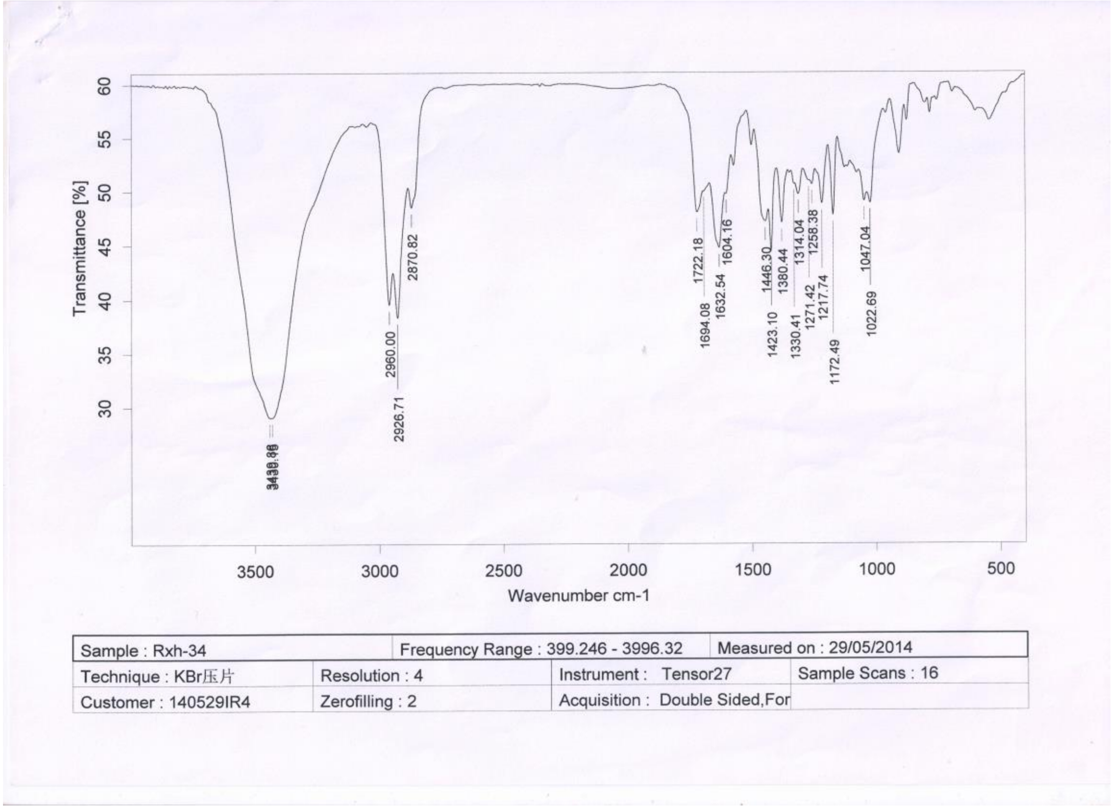

Figure S29. IR (KBr disk) spectrum of salviwardin C (**3**).

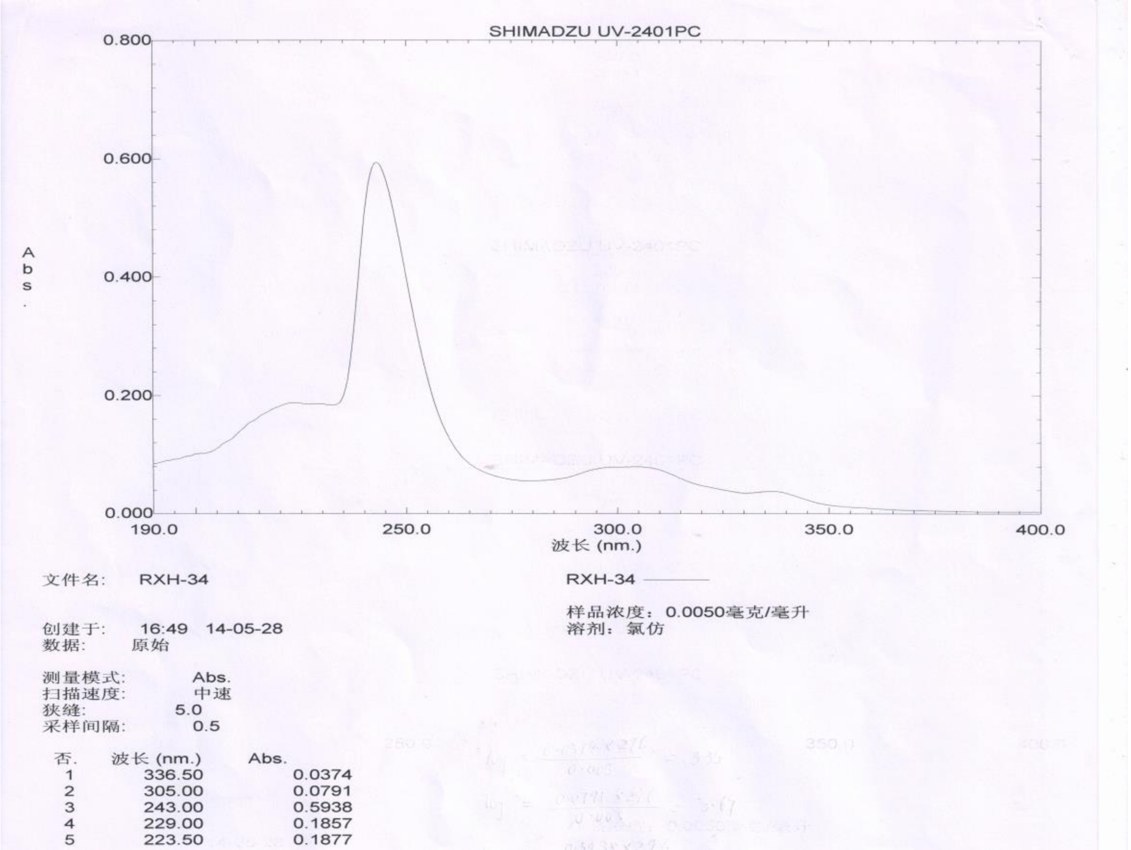

Figure S30. UV spectrum of salviwardin C (**3**).
